# MiR expression profiles of paired primary colorectal cancer and metastases by next-generation sequencing

**DOI:** 10.1038/oncsis.2015.29

**Published:** 2015-10-05

**Authors:** M Neerincx, D L S Sie, M A van de Wiel, N C T van Grieken, J D Burggraaf, H Dekker, P P Eijk, B Ylstra, C Verhoef, G A Meijer, T E Buffart, H M W Verheul

**Affiliations:** 1 Department of Medical Oncology, VU University Medical Center, Amsterdam, The Netherlands; 2 Department of Pathology, VU University Medical Center, Amsterdam, The Netherlands; 3 Department of Epidemiology and Biostatistics, VU University Medical Center, Amsterdam, The Netherlands; 4 Department of Mathematics, VU University, Amsterdam, The Netherlands; 5 Department of Pathology, Spaarne Hospital, Hoofddorp, The Netherlands; 6 Department of Surgical Oncology, Erasmus Medical Center Cancer Institute, Rotterdam, The Netherlands; 7 Department of Pathology, Netherlands Cancer Institute, Amsterdam, The Netherlands

## Abstract

MicroRNAs (miRs) have been recognized as promising biomarkers. It is unknown to what extent tumor-derived miRs are differentially expressed between primary colorectal cancers (pCRCs) and metastatic lesions, and to what extent the expression profiles of tumor tissue differ from the surrounding normal tissue. Next-generation sequencing (NGS) of 220 fresh-frozen samples, including paired primary and metastatic tumor tissue and non-tumorous tissue from 38 patients, revealed expression of 2245 known unique mature miRs and 515 novel candidate miRs. Unsupervised clustering of miR expression profiles of pCRC tissue with paired metastases did not separate the two entities, whereas unsupervised clustering of miR expression profiles of pCRC with normal colorectal mucosa demonstrated complete separation of the tumor samples from their paired normal mucosa. Two hundred and twenty-two miRs differentiated both pCRC and metastases from normal tissue samples (false discovery rate (FDR) <0.05). The highest expressed tumor-specific miRs were miR-21 and miR-92a, both previously described to be involved in CRC with potential as circulating biomarker for early detection. Only eight miRs, 0.5% of the analysed miR transcriptome, were differentially expressed between pCRC and the corresponding metastases (FDR <0.1), consisting of five known miRs (miR-320b, miR-320d, miR-3117, miR-1246 and miR-663b) and three novel candidate miRs (chr 1-2552-5p, chr 8-20656-5p and chr 10-25333-3p). These results indicate that previously unrecognized candidate miRs expressed in advanced CRC were identified using NGS. In addition, miR expression profiles of pCRC and metastatic lesions are highly comparable and may be of similar predictive value for prognosis or response to treatment in patients with advanced CRC.

## Introduction

The majority of patients with colorectal cancer (CRC) die as a consequence of metastatic disease.^1^ For patients with metastatic CRC (mCRC) combination chemotherapy with 5-fluorouracil, oxaliplatin or irinotecan and anti-vascular endothelial growth factor or anti-epidermal growth factor receptor monoclonal antibodies are available.^2^ However, 10%–25% of patients do not benefit from first-line treatment, with subsequent treatment regimens being even less effective.^3, 4, 5^ There is an urgent clinical need to develop accurate biomarkers to predict prognosis and treatment outcome of individual patients with mCRC. Currently, RAS-oncogene-testing is the only used clinical predictive molecular test for treatment of patients with mCRC.^6, 7^ Primary tumor analyses are predominantly being performed for genomic profiling, but as genomic instability is a hallmark of cancer, the genomic make-up of primary tumors and their metastases may deviate over time. In addition, adaptation of metastasized tumor cells to their specific microenvironment may lead to selection and expansion of specific clones with distinct molecular characteristics compared with the primary tumor. In previous studies, conflicting results of the genomic characteristics of both primary CRC (pCRC) and metastases from the same patients were found, varying from an almost identical make-up^8, 9, 10, 11^ to clear differences.^12, 13^

Small non-coding microRNAs (miRs) are attractive candidates to serve as biomarkers, because they display specific expression patterns and can be detected in tissues as well as in the circulating blood, as they are relatively resistant to degradation.^14, 15, 16, 17^ Recent data indicate that specific miRs have prognostic and predictive value for patients with CRC.^18, 19^ However, it is estimated that more than a third of the miRs of most cellular types are still unknown and a comprehensive comparison of miR expression profiles in mCRC is currently lacking.^20^

In this study, miR expression profiles of mCRC were robustly characterized with next-generation sequencing (NGS). Profiles of pCRC tissue and metastases from the same patients were compared, to identify whether miR expression profiles differ between pCRC and metastases. In addition, profiles of tumor tissue were compared with corresponding normal tissue, to identify tumor-specific miRs. By elucidating the miR transcriptome of mCRC, this study provides a framework for the development of miR-based biomarkers for patients with mCRC.

## Results

### The miR transcriptome of advanced CRC: identification of known and novel candidate miRs

**Figure 1:**
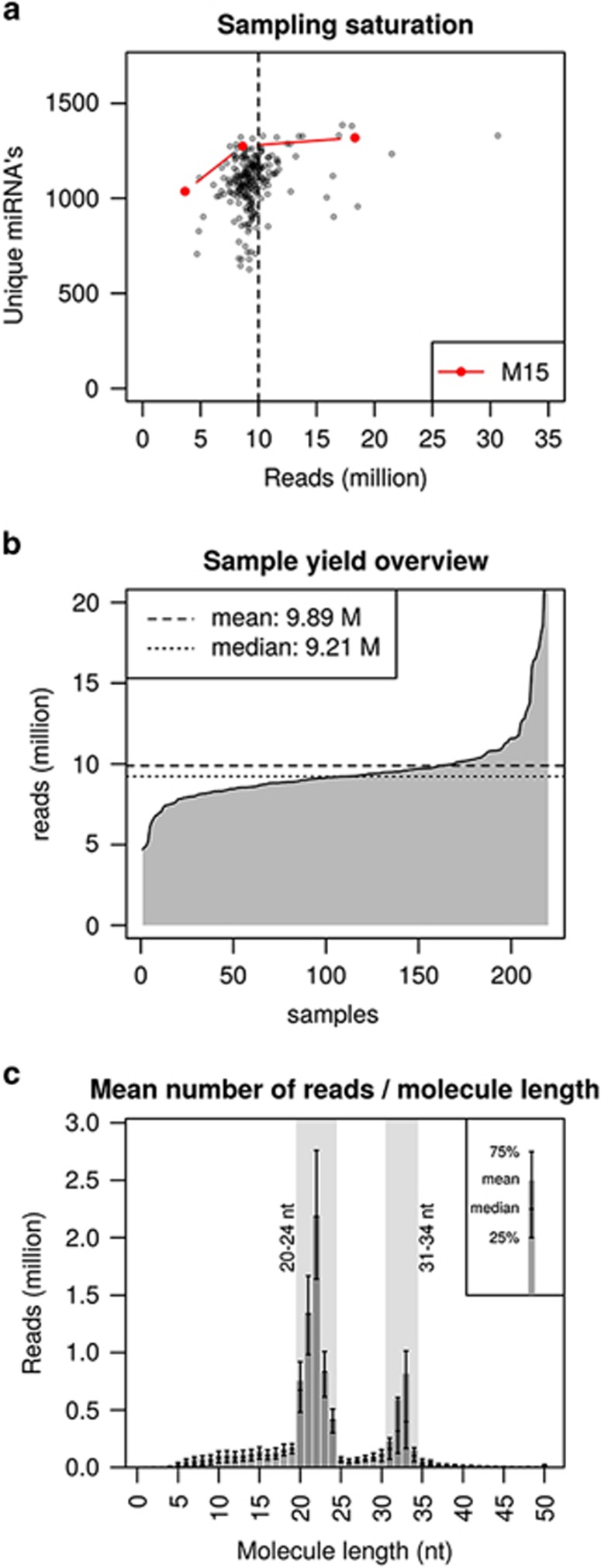
(**a**) Relationship between the numbers of raw sequence reads per sample (*x* axis) and number of unique identified miRs per sample (*y* axis) for 220 samples. Sample M15 was sequenced in triplicate at different read depths. Increasing the read depth from 3.7 to 8.6 million reads identified 238 additional unique miRs (47.9 miRs per million additional reads). Increasing the read depth from 8.6 to 18.3 million reads identified 44 additional unique miRs (4.5 miRs per million additional reads). (**b**) NGS read depth for 220 samples. Samples are shown on the *x* axis and read depth is shown on the *y* axis. Mean read depth achieved was 9.894.472 raw sequence reads per sample (dotted line). (**c**) Length distribution of the sequence reads after adapter and quality trimming in 220 samples. The *x* axis depicts the length of the sequence reads in nucleotides. The *y* axis depicts the number of reads. The bars represents the mean read count per length, the box represents the upper and lower quantiles and the median. The two length peaks represents the 20–24 nt and 31–34 nt small RNA fragments primarily selected with Illumina's TruSeq Small RNA Sample Preparation protocol. Abbreviations: M, million; nt, nucleotides.

NGS of the miR transcriptome of 220 tissue samples yielded 2 176 783 818 raw reads. Samples consisted of 126 pCRC tissue samples from patients with advanced CRC, 54 paired metastases (M), 23 paired samples with normal colorectal mucosa (PN) and 17 paired samples with normal extra-colonic tissue (MN). As demonstrated by sample M15, increasing the number of reads above ~10 million reads per sample did not result in a meaningful increase in the number of unique miRs (Figure 1a). Based on these findings, measuring ~10 million reads per sample was considered to be sufficient to analyze the miR transcriptome of mCRC, which is supported by the resulting data points from the complete study (Figure 1a). The data yield per sample ranged from 4 690 871 to 74 313 067 reads per sample with a mean of 9 894 472 reads (Figure 1b). After adapter and quality trimming, 99.5% (2 165 268 282) of initial raw reads was retained. The read length distribution after adapter and quality trimming is shown in Figure 1c. Reads of at least 18 nt, which could be mapped to the reference genome with ⩽2 mismatches, were used for the identification and quantification of the miR transcriptome of mCRC.

**Figure 2:**
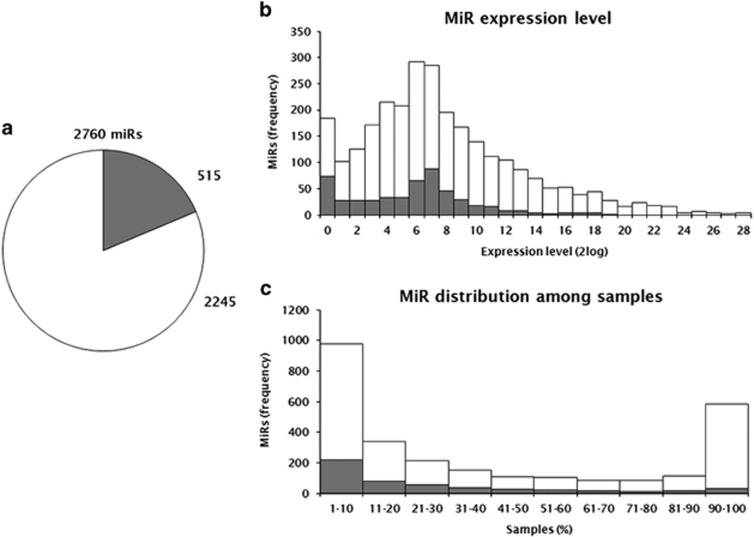
(**a**) 2760 miRs were expressed in mCRC, including 515 novel candidate miRs (shown in gray) and 2245 miRs known from miRbase v.19 (shown in white). (**b**) Log_2_ expression levels (*x* axis) of the 2760 mature miR sequences (*y* axis). Candidate miRs represented 1 567 621 reads in total (range: 1–350 911; 0.14%) and known miRs represented 1 139 882 408 reads in total (range: 1–197 979 477; 99.86%). Therefore, the higher expression levels are dominated by known miRs, whereas the candidate miRs are expressed at lower levels. (**c**) Percentage of samples (*x* axis) in which each miR is expressed (*y* axis). Nine hundred and seventy-seven miRs consisting of 217 candidate miRs and 760 known miRs were expressed in ⩽10% of the samples. Of those, 198 miRs were expressed in one sample. Five hundred and eighty-five miRs consisting of 29 candidate miRs and 556 known miRs were expressed in ⩾90% of the samples. Of those, 291 miRs were expressed in all 220 samples.

In total, 2760 unique miR sequences were observed, represented by 1 141 450 029 read counts. Five hundred and fifteen sequences represented candidate novel mature miRs and 2245 sequences corresponded to known mature miRs included in miRbase 19 (Figure 2a). The distribution of the log_2_ expression levels of the 2760 miR sequences is shown in Figure 2b. Candidate novel miRs represented 1 567 621 read counts in total (range: 1–350 911; 0.14%) and known miRs represented 1 139 882 408 read counts in total (range: 1–197 979 477; 99.86%). Of the 2760 miRs, 585 miRs were expressed in ⩾90% of the samples and 977 miRs were expressed in ⩽10% of the samples (Figure 2c). The number of miRs expressed per sample ranged from 626 to 1710 (mean 1086). The 515 novel candidate miR sequences are listed in Supplementary Table S1. These sequences were distributed throughout the genome as illustrated by their chromosomal localizations. Candidate sequences were located on all 23 chromosomes and ranged from 4 sequences on chromosome 21 to 41 sequences on chromosome 1 (Supplementary Table S1).

### Reproducibility

To check the reproducibility of the workflow, two samples were analyzed as biological triplicates and two samples were analyzed as technical duplicates. The Spearman's correlation of the miR expression levels of the biological triplicates ranged from 0.91 to 0.99 and those of the technical duplicates ranged from 0.95 to 0.98, indicating that the workflow is highly reproducible.

### MiR expression in primary CRCs and paired metastases

**Figure 3:**
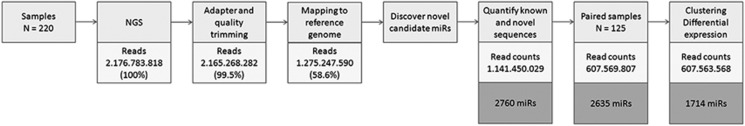
Overview of the number of raw reads, the number of reads after adapter and quality trimming, and the number of reads of at least 18 nt, which could be mapped with a maximum of two mismatches to the reference genome (browser hg 19). 2760 miRs were represented by 1 141 450 029 read counts, of which 2635 miRs were detected in at least 1 of the 125 samples used for paired sample analysis and 1714 miRs were detected in at least 3 of the 125 samples.

For the analysis of miR expression profiles of paired pCRCs and metastases, 125 samples were used corresponding to 38 individual patients with CRC (Supplementary Table S2). Of the total number of 2760 different miRs expressed in the whole data set, 2635 miRs were found in these 125 samples, representing 607 569 807 read counts. Of those, 1714 miRs were expressed in at least 3 of the 125 samples and were included for further analyses (Figure 3).

#### Unsupervised clustering

**Figure 4:**
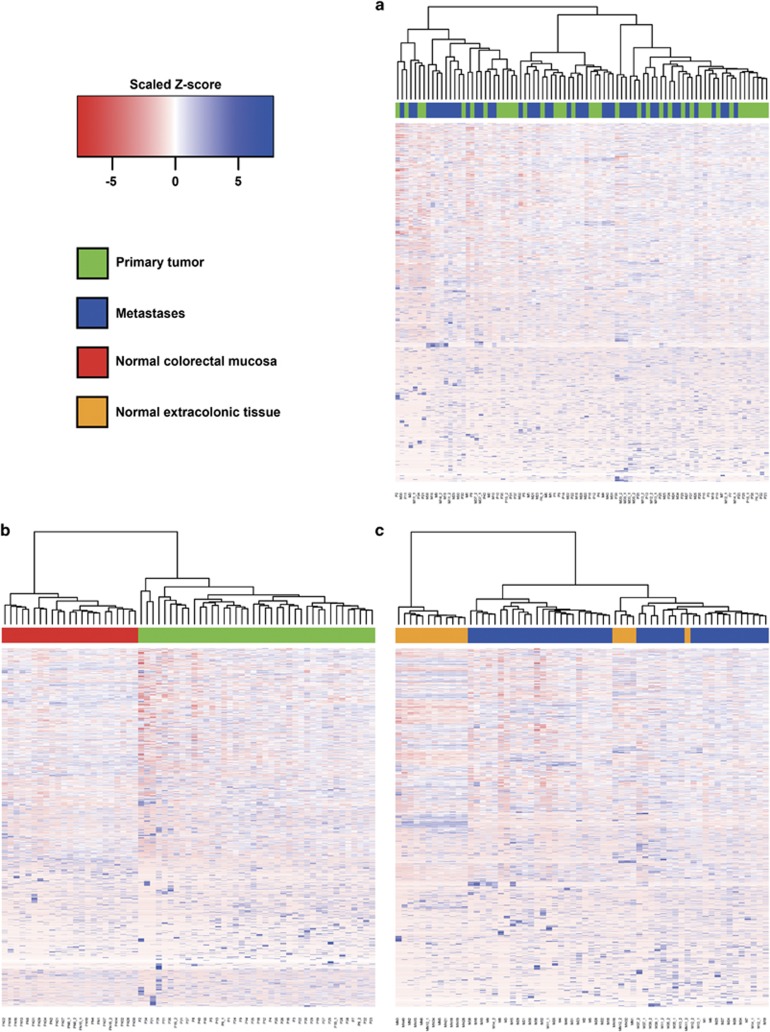
(**a**) Unsupervised clustering of log-transformed normalized miR expression levels of primary CRC samples and paired metastases of 38 patients based on 1714 miRs. Samples are shown in columns. MiRs are shown in rows. Expression levels for each miR were scaled per miR in red and blue. (**b**) Unsupervised clustering of primary CRC samples and normal colorectal epithelium. (**c**) Unsupervised clustering of metastases and normal extra-colonic tissue.

Unsupervised clustering of log-transformed normalized miR expression levels showed no clear separation of pCRC tissue with paired metastases (Figure 4a). In contrast, unsupervised clustering demonstrated complete separation of pCRC tissue from paired normal mucosa (Figure 4b). Clustering of the metastases with their paired normal extra-colonic tissue resulted in five distinct clusters. Two clusters contained only metastases and two clusters contained only normal extra colonic tissues. Normal lung epithelium (MN1, MN12_2 and MN32) clustered separately from the other normal extra colonic tissue samples. The fifth cluster contained metastases and normal gastric mucosa (MN11_2) (Figure 4c).

#### Differential expression analysis

Paired analysis of normalized expression levels of pCRC and corresponding metastatic lesions (M–pCRC) yielded 37 out of 1714 miRs (2.2%) with significant different expression levels (false discovery rate (FDR) ⩽0.10). For 29 of these 37 miRs, the difference in expression level between metastases and pCRC (|M–pCRC|) was not significantly larger than between normal extra-colonic tissue and normal colon mucosa (|MN–PN|). Therefore, the observed difference between pCRC and M was considered to be of tissue-specific origin rather than metastases-specific differential expression. After exclusion of these 29 tissue-specific miRs, 8 miRs of the initial 1714 miRs (0.5%) were expressed significantly different between pCRCs and corresponding metastases (Table 1). The eight miRs consisted of five known miRs (miR-320b, miR-320d, miR-3117, miR-1246 and miR-663b) and three novel candidate miRs (chr 1-2552-5p, chr 8-20656-5p and chr 10-25333-3p). The novel candidate miRs are located on 1q42.13, 8p23.3 and 10q26.12 (Supplementary Table S1). Of the 8 miRs, miR-320b, miR-320d and miR-1246 were expressed in all 125 samples and were expressed significantly higher in metastatic lesion compared with those in pCRC tissue, whereas miR-3117, miR-663b and 3 novel candidate miRs were expressed significantly higher in pCRC tissue compared with those in the metastatic lesion (Table 1).

### MiRs differentially expressed between tumor and normal tissue

**Figure 5:**
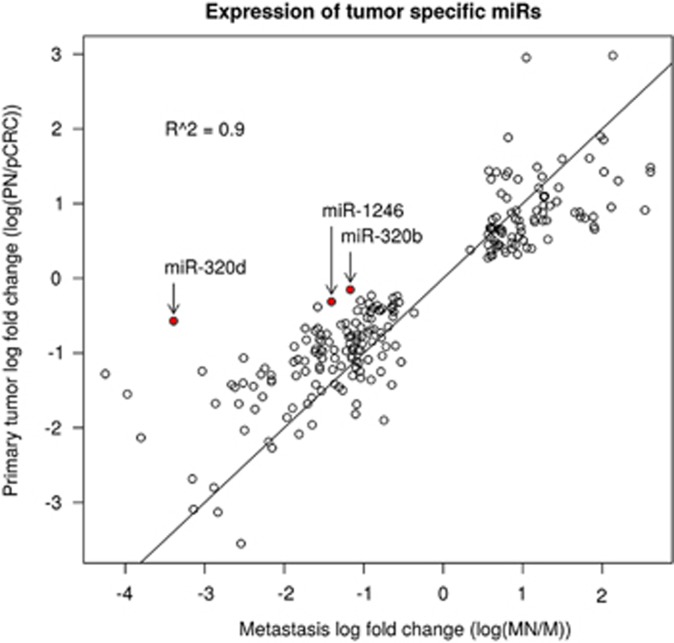
Correlation of expression level log fold change between metastasis and normal extra-colonic tissue with those between pCRC and normal colorectal mucosa of the 222 tumor-specific miRs. MiR-21-5p and miR-92a were the miRs with the highest expression in primary tumors as well as in metastases. The expression levels of the metastases-specific miRs, miR-320b, miR-320d and miR-1246 are shown in red.

Of the 1714 miRs, 222 miRs were concordantly differently expressed between metastasis and normal extra-colonic tissue (MN–M, FDR ⩽0.05) and between pCRC and normal colorectal mucosa (PN–pCRC, FDR ⩽0.05). Those miRs distinguished pCRC tissue and metastasis from normal tissue, and were considered potentially useful in diagnostic tests as well as for early detection of recurrences. One hundred and thirty-five miRs were higher expressed in the tumor tissue compared with those in the normal tissue. Of those, 121 were known mature miRs and 14 were potential novel candidate sequences (Supplementary Table S3). In addition, 87 miRs were expressed significantly lower in the tumor tissue compared with those in the normal tissue. Of those, 86 were already known mature miRs and 1 was a novel candidate sequence (Supplementary Table S4). Chromosomal location, nucleotide sequence and read count of the 15 novel candidate miRs are included in Supplementary Table S1. Figure 5 shows the correlation of the expression level fold change between MN and M with those between PN and pCRC of the 222 tumor-specific miRs. The upregulated tumor-specific miRs included miR-320b, miR-320d and miR-1246. These miRs were also expressed significantly higher in metastatic tumor tissue compared with those in pCRC tissue (Figure 5). MiR-21-5p and miR-92a were the miRs with the highest expression in pCRC as well as in metastases. Table 2 gives an overview of the upregulated tumor-specific miRs in pCRC and metastases with an overall expression level of more than 1000 (expressed as geometric mean expression level). These miRs might be the most ideal candidates for use as biomarker in clinical practice.

## Discussion

In this study it was demonstrated that the miR expression profile of metastases closely resembles that of their corresponding pCRCs. Unsupervised cluster analysis of 40 pCRCs and 45 metastases did not separate pCRCs from their metastases. Only 8 (0.5%) of the 1714 miRs used for expression analysis were expressed significantly different between pCRC and metastases. Based on these results, we expect that miR expression profiles can be further developed as predictive biomarkers for prognosis and response to treatment irrespective of a primary or secondary origin of the CRC tissue. This is of clinical significance, because tissue samples from the primary tumor are often readily available, while these are not routinely collected from metastases. There is currently no consensus whether analysis of primary tumor tissue is sufficient when analyzing the mutational status of a tumor.^11, 13, 21, 22^ Therefore, the development of miR-based biomarkers can serve as an important alternative to mutation analysis in the advanced setting.

The process of metastasis formation can be divided into specific tumor cell characteristics as follows: (1) loss of cellular adhesion, (2) increased invasiveness, (3) intravasation and survival in the vascular system, (4) extravasation, and (5) survival and proliferation at a new site.^23^ MiRs have been described to have crucial roles in acquiring these characteristics.^14, 24, 25^ and several hypotheses have been proposed to explain how tumor cell populations evolve to acquire them.^26^ Initially, the process of metastases has been seen as the final step in a sequential accumulation of (epi)genetic alterations within the site of the primary tumor.^23^ In contrast, the predestination model^27^ implies that the metastatic potential of tumor cells is already determined relatively early in carcinogenesis within the primary tumor and metastatic dissemination is not solely placed at the end of pCRC progression. The initial model and the predestination model both suggest minor genetic differences between primary tumors and metastases. According to a third model, parallel progression and evolution of primary tumors and metastases may occur at different sites.^28^ This implicates a greater disparity and variation of genetic profiles. The small differences in miR expression signatures between pCRC and metastases observed in this study suggest that the changes in miR expression levels were already present in the primary tumors and supports the predestination model.^29^ Of the eight differentially expressed miRs, miR-320b, miR-320d and miR-1246 were expressed significantly higher in the metastatic lesion compared with those in pCRC, and miR-3117, miR-663b, chr 1-2552-5p, chr 8-20656-5p and chr 10-25333-3p were expressed significantly higher in pCRC compared with those in the metastatic lesion. A role in CRC metastases formation has been proposed for miR-320b, miR-320d and miR-1246.^30, 31, 32, 33^ MiR-320b was found to be upregulated in a recent study comparing miR expression profiles of CRC patients with and without liver metastasis.^33^ Overexpression of miR320b upregulates β-catenin (CTNNB1), Neurophilin 1 (NRP1) and Ras-related C3 botulinum toxin substrate (RAC1). Interestingly, these genes are known to promote tumor metastasis.^33^ A role for miR-320d in the proliferation of CRC is suggested by *in situ* hybridization of CRC and normal colonic mucosa based on the finding that the highest expression of miR-320d was found in CRC cells and in the proliferative compartment of the colonic crypts of normal colonic mucosa.^30^ This study also demonstrated that a higher expression of miR-320d is associated with an increased recurrence free survival of stage II CRC patients. *In vitro*, circulating miR-1246 secreted by CRC cells is associated with proliferation, migration and tube formation of endothelial cells. Thereby, miR-1246 might contribute to tumor angiogenesis.^32^ Downregulation of cell adhesion molecule 1 by miR-1246 enhances migration and invasion of hepatocellular carcinoma cell lines, further suggesting a role of miR-1246 in tumor metastases formation.^31^ When these miRs involved in the process of metastasis formation are further validated, therapeutic strategies can be developed that aim at the inhibition of the oncogenic miRs or reintroduction of the tumor-suppressive miRs. The potential activity of using anti-miR oligonucleotides as a therapeutic strategy is demonstrated in a phase IIa trial for hepatitis C and is currently investigated for patients with advanced CRC as well.^34, 35, 36^

The role of miR-3117, miR-663b and the three novel candidate miRs on chr 1-2552-5p, chr 8-20656-5p and chr 10-25333-3p in the metastasizing process is unknown and functional studies have not been performed. In addition, the chromosomal locations 1q42.13, 8p23.3 and 10q26.12 on which the novel candidate miRs are located, respectively, are not known to be involved in the formation of metastases.

In this study, less differentially expressed miRs between pCRC and metastases compared with previous studies were found.^37, 38, 39^ The design of the current study has several strengths. First, fresh-frozen tissues of paired primary tumors and metastases were used. Comparing the genetic profile of metastases with unmatched primary tumors is of limited value, owing to the heterogeneity in miR expression levels between primary tumors.^24^ This is confirmed by the unsupervised clustering analysis, demonstrating a large heterogeneity in miR expression levels between tumors of different patients. Second, 89% of the tumor samples yielded a tumor cell content of more than 70%, thereby minimizing the influence of the expression of non-tumorous miRs. By including the miR expression profiles of adjacent normal tissue in the analysis as well, the influence of non-tumorous miRs on the differential expression analysis between pCRC and metastases was further minimized. Third, the amount of measured miRs was more than doubled compared with previously published studies identifying the miR transcriptome of CRC.^40, 41, 42^ Prior reports comparing miR expression in mCRC used probe-based methodologies,^37, 38, 39^ which, by definition, are restricted to the detection and profiling of the known miR molecules. Recent studies using NGS-based methodologies used sequencing depths varying between one million and three million reads per sample.^42, 43^ However, the high dynamic expression range of miRs can result in a profile that is dominated by a few highly expressed miRs, which makes it difficult to detect low-expressed miRs.^44, 45^ Therefore, the preferred read depth was first identified at ~10 million reads per sample. Owing to the overrepresentation of liver metastases compared with the other locations, it was not possible to analyse whether the location of metastases had an effect on differential expression; for example, whether there were miR expression profiles specific for the location of the metastases. Likewise, it was not possible to include the organ of metastasis for the MN–PN comparison because of the overrepresentation of normal epithelium of the liver. In addition, miRs that are not phylogenetically conserved might have been missed by using miRdeep2 as prediction algorithm to identify novel candidate miR sequences.

In contrast to the minor differences observed between pCRC and metastatic tumor tissue, 222 tumor-specific miRs were observed with a significantly different expression profile in both pCRC as well as metastases compared with its adjacent normal tissue. We hypothesize that upregulated tumor-specific miRs might yield the potential to assist in early detection or recurrence of pCRC or distant CRC metastases by measuring the circulating levels of these miRs. Mitchell *et al.*^17^ were the first to demonstrate that tumor-derived circulating miRs had the potential to detect solid cancers and blood-based miR profiles specific for cancers and non-cancer diseases have been established since.^46^ MiR-21 and miR-92a were the two highest expressed discriminatory miRs in the current study. Strikingly, these two miRs were recently identified for having potential as circulating biomarker for early detection and screening of CRC.^47, 48, 49, 50^ Furthermore, both miR-92a and miR-21expression were shown to correlate with mCRC and regulate invasion and metastases by inhibiting phosphatase and tensin homolog.^51, 52, 53^ Five metastases-specific miRs were not represented in the 222 tumor-specific miRs, because for those miRs the difference in expression level between tumor tissue and adjacent normal tissue was not significant for both pCRC and metastases.

In summary, NGS was used to analyze miR expression profiles of mCRC including both known and novel candidate miRs. MiR expression profiles of pCRC and metastases were highly comparable and may therefore be of similar predictive value for prognosis or treatment response for patients with mCRC. We foresee that in the coming years detection of specific low-abundant miRs might be performed using targeted sequencing, looking in depth at the expression level of a selected number of miRs. This increased sensitivity will make it possible to include important discriminatory low-abundant miRs in a prediction algorithm to select patients in clinical practice.

## Materials and Methods

### Patients and tumor samples

Two hundred and twenty fresh-frozen tissue samples resected between 1997 and 2012 were collected, to characterize the miR transcriptome of mCRC. Samples consisted of 126 pCRC tissues samples, 54 metastases (M), 23 samples with normal colorectal mucosa (PN) and 17 samples with normal extra-colonic tissue (MN). The metastatic tissue specimens consisted of 23 liver, 5 lung, 6 ovarian and 9 peritoneal metastases, and 9 metastases in the distant lymph nodes, 1 metastasis in the stomach and 1 in the thoracic wall. Samples were collected from the archives of the VU University Medical Center of Amsterdam, the Spaarne Hospital of Hoofddorp and the Erasmus University Medical Center of Rotterdam, according to the ethical guidelines of these hospitals. Samples from patients with neoadjuvant radiotherapy or systemic therapy within 6 months before resection of the primary tumor were excluded.

Samples included 125 paired tissue samples of 38 consecutive patients of which pCRC tissue samples as well as corresponding synchronous or metachronous metastases were directly frozen after surgery. An overview of tumor and patient characteristics of the paired tissue samples is given in Supplementary Table S2. The paired tissue samples consisted of 40 pCRC samples, 45 metastases, 23 samples with normal colorectal mucosa and 17 samples with normal extra-colonic tissue. Two pCRC samples were microsatellite instable. The metastatic tissue specimens consisted of 20 liver, 4 lung, 6 ovarian and 5 peritoneal metastases, and 8 metastases in the distant lymph nodes. One metastasis was located in the stomach and 1 in the thoracic wall. The normal extra-colonic tissue samples included 12 samples with liver tissue, 3 samples with lung tissue, 1 sample with ovarian tissue and 1 sample with gastric mucosa. From seven patients two different metastatic localizations were included and from one patient two independent primary tumors were included. From one patient, material from the original tumor was lacking and tumor material of the local recurrence was used instead. From another patient, material from the primary tumor, the local recurrence and the metastatic lesion was included. In 12 cases, systemic therapy was given between resection of the primary tumor and the subsequent resection of a metastasis, and 4 cases were within 6 months before resection of the metastases. All tumors were classified according to the WHO classification for colorectal carcinomas.^54^ Normal colorectal mucosa and normal extra-colonic tissues were histologically classified as cancer-free.

### RNA isolation

Four-micrometer sections were made of each tumor sample, stained with hematoxylin and eosin and evaluated by a gastro-intestinal (GI) pathologist (NCTvG or GAM). Tumor areas with the highest tumor cell density were selected and the remaining tissue was macrodissected and removed from the tissue specimen. A new 4-μm hematoxylin and eosin section was made and evaluated for tumor cell content. Macrodissection was repeated until the tumor cell density could not be further improved. After macrodissection, 10–40 (depending on the tumor surface area) 25 μm slides were cut and directly frozen in the liquid nitrogen. Sandwich hematoxylin and eosin sections were made and independently evaluated for tumor content. Of all 180 tumor samples (126 primary tumors and 54 metastases), 160 (89%) yielded at least 70% tumor cells. The 20 tumor samples containing <70% tumor cells (range: 35%–65%) were all classified as mucinous tumors or showed a high percentage of inflammatory cells. Of the 85 paired tumor samples (40 pCRC samples and 45 metastases) used for the miR expression analysis between primary tumors and metastases, 74 (87%) contained at least 70% tumor cells (Supplementary Table S2). Sandwich hematoxylin and eosin slides of the normal tissue samples were classified as 100% cancer free. Total RNA was isolated using TRIzol (Invitrogen, Carlsbad, CA, USA) and RNA quantity was determined with a Nanodrop 2000 (Thermo Scientific, Waltham, MA, USA). To optimize the isolation of small RNA species, isopropanol volume was 50% increased and 75% ethanol was used two times as wash solution.

### Next-generation sequencing

Illumina's TruSeq Small RNA Sample Preparation protocol (Illumina Inc., San Diego, CA, USA) was used to prepare the cDNA libraries with 1 μg RNA input. Forty-eight unique barcode sequences were applied for simultaneous analysis of multiple samples. Sequence library yield was assessed using the Agilent 2100 Bioanalyzer (Agilent Technologies, Santa Clara, CA, USA) with DNA1000 chips before sequencing. The library was loaded onto an Illumina cluster station (Illumina Inc., San Diego, CA, USA) and sequenced using Illumina's High Seq 2000 (Illumina Inc., San Diego, CA, USA). The optimal read depth to analyse the miR transcriptome of CRC tissue was determined at 10 million reads per sample (Figure 1a).

### Data filtering

Several data filtering steps were performed after obtaining the raw reads. First, the FASTQ Quality Trimmer (http://hannonlab.cshl.edu/fastx_toolkit) was applied to trim the 3′-end of the reads from nucleotides with a Phred-scaled quality score below 30, corresponding to a >99.9% probability of a correctly identified base. Second, the 3′-ends of the reads were clipped for adaptor sequences. Third, reads with identical sequences were counted and collapsed resulting in only unique sequences to reduce the storage and computation requirements. Finally, each unique sequence was mapped to the reference genome (browser hg19) and alignments of at least 18 nt and a maximum of 2 mismatches were retained. Genome data have been deposited at the European Genome-phenome Archive (http://www.ebi.ac.uk/ega/), which is hosted at the European Bioinformatics Institute (EBI), under accession number EGAS00001001127.

### Identification of novel candidate miRs

The miRDeep2 package was used to identify novel candidate miRs in the obtained deep sequencing data,^55^ as this method was found to be most suitable for identifying novel miR candidates.^56^ This package uses a probabilistic model of miR biogenesis to score compatibility of the position and frequency of sequenced RNA with the secondary structure of the miR precursor.^55^ The majority of miRs are transcribed as long primary transcripts from which one or more ~70-nt-long hairpin precursors (pre-miRs) are cleaved out by the Drosha endonuclease.^57^ Therefore, for each read, potential precursor sequences were retrieved from both genome contigs, one including 70 nt upstream and 20 nt downstream flanking sequence, and one including 20 nt upstream and 70 nt downstream flanking sequence. For each candidate pre-miR sequence, the potential secondary structure was predicted. Based on those predicted secondary structures of the potential precursor sequences, the thermodynamic energy to fold these precursors and the conservation among three species (chimpanzee, mouse and rat), predictions for each sequence read were made. The presence of multiple sequenced RNAs corresponding to the mature miR, the presence of the complementary strand of the mature miR and the presence of the loop of the precursor in the sequencing data were used as a support to identify a sequence as novel candidate miR. Reads from all 220 samples were pooled during the identification of known and novel miRs, as novel candidate miRs can be more accurately predicted by detecting both the −5p and –3p sequences in multiple independent samples. In order to exclude sequences originating from repetitive elements, reads that aligned to more than five positions in the genome were excluded from further analysis. In addition, sequences that could be mapped to other known non-coding RNAs or sequences within coding regions were excluded. For each analysis, the lowest cutoff score that yielded a signal-to-noise ratio of 5:1 or higher was used (Friedlander, personal correspondence). The signal-to-noise ratio was estimated as the number of total miRs (novel candidate miRs and known miRbase v.19 miRs) divided by the estimated total number of false-positive novel candidate miRs. The number of false positives was calculated for a given cutoff point by permutation.

### Quantification of the miR transcriptome of mCRC

Sequencing reads were quantified by mapping them against precursor sequences from mirbase v.19 and the novel predicted precursor sequences resulting from the miRDeep2 analyses. A sequencing read (up to one mismatch was allowed) was assumed to represent a sequenced mature miR if it aligned within the same position on the precursors as the known or predicted mature –3p or –5p sequence (no mismatch was allowed). A small window of 2 nt upstream and 5 nt downstream around the annotated mature miR in its precursor was allowed, because sequencing reads originating from true miRs can be subjected to untemplated nucleotide addition and inaccurate Dicer processing. Reads that map equally well to the positions of multiple mature miRs were added to the read counts of those mature miRs. However, miRs mapping to an unrelated precursor were removed from further analysis. Read counts of identical mature miRs mapping to related precursors (for example, hsa-mir-7-1, hsa-mir-7-2 and hsa-mir-7-3) were averaged.

### Statistical analysis

Unsupervised clustering and pair-wise comparisons were performed on miRs with expression in at least three samples. Normalization was done using edgeRs TMM method.^58^ Unsupervised clustering was done using Euclidean distance between the log_2_ of normalized expression levels and using Ward's minimum variance linkage across samples. The cluster analysis was performed in R using the gplots package, version 2.16.0. Pair-wise comparisons were performed using the R-package ShrinkBayes, version 2.8,^59^ which is accessible on http://www.few.vu.nl/~mavdwiel/ShrinkBayes.html. To account for multiple testing, an FDR was estimated using the Bayesian FDR estimate.^60^ The mean expression value was expressed as a geometric mean value, which is a conventional summary for (skewed) count data. For the comparison of pCRC with metastases, miRs were selected to be significantly differentially expressed if FDR ⩽0.10. Compared with the analysis of tumor-specific miRs, a less strict FDR for these comparisons was used to decrease the number of false-negative miRs. To correct for miR expression in non-tumorous tissue, it was determined whether the difference in expression level between pCRC and M was significantly larger (one sided test) than between normal colorectal mucosa (PN) and normal tissue of the organ of metastases (MN). To account for potential confounders on differences in miR expression, the following additional covariates in the regression models were included: organ of metastasis, time between the resection of pCRC tissue and the metastatic tissue, and the use of chemotherapy in the time period between the resections. If the time between resection of pCRC and metastases was <90 days, or if the metastases were resected before resection of the pCRC, this pair of tumor tissue was considered to be synchronously metastasized (no time between the resections). If a patient had two pCRCs, the mean time between the resections of those pCRCs with the metastasis was used for analysis. If a patient had two metastases resected, both paired comparisons were included. Organ of metastasis was only included as a covariate for the M–pCRC comparison and not for the MN–PN comparison, because normal epithelium of the liver was overrepresented compared with the other organs.

To determine which miRs were tumor specific, it was analyzed which miRs were differently expressed between M and MN samples, and were concordantly differentially expressed between pCRC and PN samples. Given the large number of differential miRs for these comparisons, a more restrictive FDR cutoff was used to minimize the number of false-positive miRs (FDR ⩽0.05).

## Figures and Tables

**Table 1:** Colorectal cancer metastases-specific miRs

*miRNA*	*Geometric mean*^a^				*M–pCRC*		*MN–PN*		*(|M–pCRC|–|MN–PN|)*	
	*M (*n=*45)*	*pCRC (*n=*40)*	*MN (*n=*17)*	*PN (*n=*23)*	*FDR*	*Log fold change*	*FDR*	*Log fold change*	*FDR*	*Log fold change*
hsa-miR-1246	293.0 (100)	79.0 (100)	67.9 (100)	56.7 (100)	0.017	0.84	0.032	−0.91	0.000	2.32
hsa-miR-320b	330.0 (100)	220.0 (100)	202.0 (100)	214.0 (100)	0.043	0.45	0.112	−0.16	0.002	0.80
hsa-miR-320d	41.0 (100)	27.0 (100)	20.9 (100)	20.7 (100)	0.091	0.40	0.277	−0.07	0.005	0.82
hsa-chr1_2552-5p	1.6 (73)	1.8 (80)	2.5 (59)	3.8 (91)	0.035	−0.41	0.490	0.06	0.046	−0.85
hsa-miR-3117-3p	4.8 (84)	11.3 (98)	7.0 (88)	2.4 (83)	0.072	−0.46	0.497	0.04	0.006	−1.11
hsa-chr10_25333-3p	0.2 (18)	0.7 (60)	0.8 (59)	0.8 (65)	0.085	−0.54	0.460	0.05	0.012	−1.30
hsa-miR-663b	0.8 (49)	1.9 (88)	2.9 (65)	1.8 (70)	0.095	−0.56	0.172	0.30	0.003	−1.62
hsa-chr8_20656-5p	1.0 (56)	2.7 (93)	2.9 (65)	0.7 (57)	0.064	−0.62	0.118	0.46	0.009	−1.63

Abbreviations: FDR, false discovery rate; M, metastases; miR, microRNA; MN, normal extracolonic tissue; pCRC, primary colorectal cancer; PN, normal colorectal mucosa.

Fold change is noted as natural logarithm.

FDR was estimated using the Bayesian FDR estimate.

Overview of the eight metastases-specific miRs, including the mean expression values, percentage of samples in which these miRs are expressed, FDRs and log fold changes.

a Expression level is noted as mean geometic value. In brackets are the percentage of samples that expressed the mature miR.

**Table 2:** Tumor-specific miRs with an overall expression of more than 1000 (expressed as geometric mean expression level) in pCRC and metastases

*miRNA*	*Geometric mean*^a^				*FDR*		*Log fold change*	
	*M (*n=*45)*	*MN (*n=*17)*	*pCRC (*n=*40)*	*PN (*n=*23)*	*MN vs M*	*PN vs pCRC*	*MN vs M*	*PN vs pCRC*
hsa-miR-21-5p	256 999	156 999	232 999	128 999	0.0000	0.0000	−1.18	−0.79
hsa-miR-92a-3p	99 499	73 699	80 099	40 799	0.0006	0.0000	−0.60	−0.90
hsa-miR-182-5p	36 199	9599	29 399	9189	0.0000	0.0000	−2.29	−1.28
hsa-miR-21-3p	14 899	8409	13 099	5049	0.0000	0.0000	−1.10	−1.11
hsa-miR-25-3p	10 099	8349	9449	6099	0.0148	0.0000	−0.37	−0.46
hsa-miR-93-5p	6199	4159	5359	3809	0.0000	0.0000	−0.96	−0.53
hsa-miR-98-5p	5709	4489	6519	4219	0.0013	0.0408	−0.63	−0.26
hsa-miR-183-5p	3919	980	4259	842	0.0000	0.0000	−2.66	−1.42
hsa-miR-181c-5p	3839	2649	3109	2519	0.0007	0.0004	−0.91	−0.54
hsa-miR-19b-3p	3819	2739	2679	1859	0.0021	0.0002	−0.69	−0.65
hsa-miR-20a-5p	3629	2439	3069	1829	0.0000	0.0000	−1.02	−0.88
hsa-miR-92b-3p	3529	2329	5749	3049	0.0156	0.0166	−0.65	−0.39
hsa-miR-23a-3p	3519	2829	3419	2859	0.0036	0.0191	−0.58	−0.24
hsa-miR-222-3p	3419	1809	3609	2429	0.0011	0.0012	−0.99	−0.44
hsa-miR-532-5p	2819	1769	2389	1719	0.0000	0.0119	−0.83	−0.36
hsa-miR-17-5p	2549	1599	2139	1139	0.0000	0.0000	−1.12	−0.92
hsa-miR-335-3p	1679	1079	1809	695	0.0000	0.0000	−1.15	−1.04
hsa-miR-941	1539	1029	1749	923	0.0000	0.0189	−0.92	−0.45

Abbreviations: FDR, false discovery rate; M, metastases; miR, microRNA; MN, normal extracolonic tissue; pCRC, primary colorectal cancer; PN, normal colorectal mucosa.

MiRs were higher expressed in both pCRC and metastases compared with normal adjacent tissue.

a Expression level is noted as mean geometic value. Fold change is noted as natural logarithm. FDR was estimated using the Bayesian FDR estimate.
