# Supplementary material for: MiR expression profiles of paired primary colorectal cancer and metastases by next-generation sequencing
Source: Oncogenesis. 2015 Oct 5;4(10):e170–. doi: 10.1038/oncsis.2015.29 (PMC4632090; doi:10.1038/oncsis.2015.29)
Supplement: Supplementary Information [file oncsis201529x1.doc]

**Data Supplement, online only.**

**Supplementary Table S1.** Chromosomal localization, nucleotide sequence and read count of the 515 novel candidate miR sequences. Three candidate sequences were differentially expressed between primary tumors and metastases and are shown in bold. Fifteen candidate sequences were differentially expressed between tumor tissue and non-tumorous tissue and are shown in italic.

| **miRNA** | **Sequence** | **Chromosomal location** | **Read count** |
| --- | --- | --- | --- |
| hsa-chr1_1039-3p | uaauuucuguuuuucucuuaca | chr1:43892704-43892726:+ | 75 |
| hsa-chr1_1039-5p | gaggaggggaacaggggu | chr1:43892662-43892680:+ | 1 |
| hsa-chr1_1361-3p | uugaguuacuuugccuuauccc | chr1:87517032-87517054:+ | 112 |
| hsa-chr1_1361-5p | gaugaggcagaguaacucaca | chr1:87516996-87517017:+ | 11 |
| hsa-chr1_1375-3p | aggguuccgccggccacc | chr1:91172691-91172709:+ | 83 |
| hsa-chr1_1375-5p | guggccgaggcggcggcu | chr1:91172622-91172640:+ | 2 |
| hsa-chr1_1439-3p | cuugagacucugggucagucu | chr1:95620430-95620451:+ | 335 |
| hsa-chr1_1439-5p | gacugacccagagucucaagc | chr1:95620395-95620416:+ | 10 |
| hsa-chr1_1463-3p | ucuuccagauguuacugacugc | chr1:102074847-102074869:+ | 83 |
| hsa-chr1_1463-5p | uaguuaguaacaucuggaagag | chr1:102074810-102074832:+ | 10 |
| *hsa-chr1_2265-3p* | *cuugggcuccugcugcgcgcagc* | *chr1:199998392-199998415:+* | *1080* |
| hsa-chr1_2265-5p | acgcgcgcucgggggcucg | chr1:199998357-199998376:+ | 4 |
| hsa-chr1_2353-3p | uuagcucccucuucccauguuc | chr1:204965493-204965515:+ | 46 |
| hsa-chr1_2353-5p | gcugggaagguggugcugcuga | chr1:204965456-204965478:+ | 3 |
| hsa-chr1_2552-3p | gcuggcgccgccggagcg | chr1:228871285-228871303:+ | 1 |
| **hsa-chr1_2552-5p** | **uccacggcgcucggaccg** | **chr1:228871239-228871257:+** | **1065** |
| hsa-chr1_2687-3p | auucaucagccgucagga | chr1:244256481-244256499:+ | 302 |
| hsa-chr1_2687-5p | auugaggguggugaggcu | chr1:244256419-244256437:+ | 1 |
| hsa-chr1_3104-3p | cuucagcugcagaacucucagu | chr1:23756052-23756074:- | 110 |
| hsa-chr1_3104-5p | cccuguggcuggaucugagug | chr1:23756086-23756107:- | 2 |
| hsa-chr1_3403-3p | ucugugucuccaguggcuaggc | chr1:44748959-44748981:- | 1 |
| hsa-chr1_3403-5p | auggccaucuggaucacagaga | chr1:44748997-44749019:- | 45 |
| hsa-chr1_3720-3p | cuugagacucugggucagucu | chr1:95620393-95620414:- | 335 |
| hsa-chr1_3720-5p | gacugacccagagucucaagc | chr1:95620428-95620449:- | 10 |
| hsa-chr1_3781-5p | uccuggagcuccuggacu | chr1:109788887-109788905:- | 36 |
| hsa-chr1_3875-5p | ucucugggccugugucuu | chr1:142850116-142850134:- | 156 |
| hsa-chr1_3927-3p | uccacuuuugggguucagagau | chr1:146629730-146629752:- | 16 |
| hsa-chr1_3927-5p | cacugaauuccauuuguggacu | chr1:146629765-146629787:- | 262 |
| hsa-chr1_4097-5p | acagcugaagcauggacu | chr1:151702273-151702291:- | 38 |
| hsa-chr1_4286-3p | agcggaacuugaggagccgaga | chr1:161416444-161416466:- | 162 |
| hsa-chr1_4286-5p | uugggcuccacgggugucagcgg | chr1:161416482-161416505:- | 9 |
| hsa-chr1_4288-3p | agcggaacuugaggagccgaga | chr1:161423824-161423846:- | 162 |
| hsa-chr1_4288-5p | uugggcuccacgggugucagcgg | chr1:161423862-161423885:- | 9 |
| hsa-chr1_4290-3p | agcggaacuugaggagccgaga | chr1:161431235-161431257:- | 162 |
| hsa-chr1_4290-5p | uugggcuccacgggugucagcgg | chr1:161431273-161431296:- | 9 |
| hsa-chr1_4292-3p | agcggaacuugaggagccgaga | chr1:161438615-161438637:- | 162 |
| hsa-chr1_4292-5p | uugggcuccacgggugucagcgg | chr1:161438653-161438676:- | 9 |
| hsa-chr1_4460-3p | aacccaccacugccacca | chr1:181513728-181513746:- | 1252 |
| hsa-chr1_4460-5p | gaaugaaggagggggaga | chr1:181513790-181513808:- | 2 |
| hsa-chr1_687-3p | uaauguaguugccacuaggaga | chr1:20236985-20237007:+ | 94 |
| hsa-chr1_687-5p | uuuaguggcaacagcuuugaac | chr1:20236917-20236939:+ | 6 |
| hsa-chr2_5137-3p | gguccagaucagagagac | chr2:21022975-21022993:+ | 51 |
| hsa-chr2_5137-5p | uucuuacuggcuuggagc | chr2:21022953-21022971:+ | 20 |
| hsa-chr2_5318-3p | agcauuucagauuucagguuu | chr2:46575991-46576012:+ | 108 |
| hsa-chr2_5318-5p | aucugaaauuugaaauggucc | chr2:46575933-46575954:+ | 258 |
| hsa-chr2_5338-3p | cgagggccgucccggggagca | chr2:47596900-47596921:+ | 5 |
| hsa-chr2_5338-5p | ugcuccggcucaggcccuccgc | chr2:47596864-47596886:+ | 72 |
| hsa-chr2_5510-3p | ucuccagcaaacugggacagu | chr2:71754024-71754045:+ | 2 |
| hsa-chr2_5510-5p | auccuagcuugccugagacug | chr2:71753958-71753979:+ | 108 |
| hsa-chr2_5799-3p | auuuuuucuggagauucuguuc | chr2:103679083-103679105:+ | 88 |
| hsa-chr2_5846-3p | ccucagccacugcugacaccagg | chr2:111893001-111893024:+ | 5 |
| hsa-chr2_5846-5p | uuguguccaguuguugggggag | chr2:111892962-111892984:+ | 66 |
| hsa-chr2_5863-3p | ccuuggacaucugcucuuccaga | chr2:113326301-113326324:+ | 87 |
| hsa-chr2_5863-5p | augguaagaguaaauguguaacc | chr2:113326260-113326283:+ | 6 |
| hsa-chr2_5950-5p | gcacucuggacagacugcc | chr2:128399316-128399335:+ | 214 |
| hsa-chr2_6018-3p | caggcaugacaaccucauacu | chr2:133191344-133191365:+ | 5 |
| hsa-chr2_6018-5p | ugugagguugucaugccugcu | chr2:133191304-133191325:+ | 1293 |
| hsa-chr2_6230-3p | ugggcccugccaggcuugccu | chr2:176966747-176966768:+ | 12 |
| hsa-chr2_6230-5p | caugucuggccugucccagug | chr2:176966711-176966732:+ | 43 |
| hsa-chr2_7356-3p | ugcccaggggcugugagcc | chr2:85060871-85060890:- | 491 |
| hsa-chr2_7356-5p | ugacagccccugggcacuccu | chr2:85060901-85060922:- | 15 |
| hsa-chr2_7480-3p | ugcccggcgggcgccggc | chr2:96931076-96931094:- | 618 |
| hsa-chr2_7480-5p | cgggcggccccgggcaugu | chr2:96931115-96931134:- | 2 |
| hsa-chr2_7678-3p | ccuggugcucugccccucagg | chr2:127806208-127806229:- | 59 |
| hsa-chr2_7678-5p | cugugggguggagccccuugcuc | chr2:127806241-127806264:- | 4 |
| hsa-chr2_7825-3p | ccuggugaugguagcugaau | chr2:133670659-133670679:- | 1 |
| hsa-chr2_7825-5p | auucagcuacgaucaccagggca | chr2:133670696-133670719:- | 79 |
| hsa-chr2_7838-3p | aauagugucuagaauaucuuga | chr2:136092843-136092865:- | 124 |
| hsa-chr2_7838-5p | aagauauucuagacacuauucu | chr2:136092876-136092898:- | 18 |
| hsa-chr2_7904-3p | cuccguccuccuccuccccc | chr2:153574151-153574171:- | 4107 |
| hsa-chr2_7904-5p | guagaguuuucccgacggaggacu | chr2:153574186-153574210:- | 1 |
| hsa-chr2_8043-3p | caaaguuuaagauccuugaugu | chr2:189162241-189162263:- | 3 |
| hsa-chr2_8043-5p | uucaaggaucuuaaacuuugccu | chr2:189162275-189162298:- | 612 |
| hsa-chr3_10583-3p | auggccgugcucugucagag | chr3:141087033-141087053:- | 6 |
| hsa-chr3_10583-5p | agacagauucaacaaggc | chr3:141087090-141087108:- | 98 |
| hsa-chr3_10607-3p | uagguuguaggaugcuaaac | chr3:143501969-143501989:- | 4 |
| hsa-chr3_10607-5p | uguuuagcauccuguagccugc | chr3:143502000-143502022:- | 118 |
| hsa-chr3_8468-3p | caaaaaccguaauuacuuuugu | chr3:4493154-4493176:+ | 25 |
| hsa-chr3_8468-5p | aaaaguaaucgcggucuuugcc | chr3:4493118-4493140:+ | 608 |
| hsa-chr3_8528-3p | cugcugagggcuucaguu | chr3:10661403-10661421:+ | 1 |
| hsa-chr3_8528-5p | cugcagcuuucagaccug | chr3:10661336-10661354:+ | 168 |
| hsa-chr3_8873-3p | ccuucucgagccuugagugugc | chr3:52428009-52428031:+ | 43 |
| hsa-chr3_8873-5p | ggcucaagguucaagaaggc | chr3:52427971-52427991:+ | 11 |
| *hsa-chr3_8875-3p* | *aacaggccuugcucugcucacaga* | *chr3:52557420-52557444:+* | *813* |
| hsa-chr3_8875-5p | ugugggcagagcagagccugca | chr3:52557372-52557394:+ | 57 |
| hsa-chr3_8898-3p | ugugguggcugcugcuggugcc | chr3:53797112-53797134:+ | 14 |
| hsa-chr3_8898-5p | cacaggcaggagaccccacagc | chr3:53797073-53797095:+ | 182 |
| hsa-chr3_8988-3p | cuuuuccucagagcaggg | chr3:78712870-78712888:+ | 1 |
| hsa-chr3_8988-5p | gagggcugugguaaaagc | chr3:78712826-78712844:+ | 50 |
| hsa-chr3_9077-3p | acagguccuaagagacugcauc | chr3:112455987-112456009:+ | 65 |
| hsa-chr3_9077-5p | uguggccucuaagaacccgcucu | chr3:112455951-112455974:+ | 11 |
| hsa-chr3_9237-3p | ucugcugagaguuucugacuga | chr3:133785773-133785795:+ | 76 |
| hsa-chr3_9237-5p | gucaaaaacucucaguggacu | chr3:133785735-133785756:+ | 5 |
| hsa-chr3_9335-3p | cauccaccuaugucuucugcagc | chr3:148601378-148601401:+ | 48 |
| hsa-chr3_9335-5p | aggcaggagcuauauggauggg | chr3:148601340-148601362:+ | 7 |
| hsa-chr3_9795-5p | uccccagagcccggacug | chr3:14446816-14446834:- | 38 |
| hsa-chr3_9914-3p | cugugugugucugaggcu | chr3:38631113-38631131:- | 37 |
| hsa-chr4_10935-3p | ugccucccaccccuuccccagu | chr4:1807757-1807779:+ | 95 |
| hsa-chr4_10935-5p | agcggggagagguggagaggcuucagccc | chr4:1807728-1807757:+ | 3 |
| hsa-chr4_11310-3p | ccucguuugccucgcgcc | chr4:75858380-75858398:+ | 1 |
| hsa-chr4_11310-5p | cacgcagcugacggagcug | chr4:75858350-75858369:+ | 923 |
| hsa-chr4_11627-3p | uuccccgcuuccccccuagggg | chr4:141073481-141073503:+ | 87 |
| hsa-chr4_11627-5p | caaagggggugagcuggggagag | chr4:141073441-141073464:+ | 11 |
| hsa-chr4_11702-3p | agugccauugacuuagagucacc | chr4:154095381-154095404:+ | 3 |
| hsa-chr4_11702-5p | gauucuaagucaauggcacug | chr4:154095343-154095364:+ | 20 |
| hsa-chr4_11713-3p | uugagaauuaugaugugccu | chr4:156810167-156810187:+ | 52 |
| hsa-chr4_11801-3p | auaacucuggccccaggcacgu | chr4:185306128-185306150:+ | 112 |
| hsa-chr4_11801-5p | gcagccuggggacagagg | chr4:185306081-185306099:+ | 2 |
| hsa-chr4_12093-5p | cuguggggaucuggcacu | chr4:52844353-52844371:- | 89 |
| hsa-chr4_12178-3p | ugugcaggcaugcaguuuauguu | chr4:76895034-76895057:- | 152 |
| hsa-chr4_12178-5p | cauaaacugcaugccugcacacc | chr4:76895070-76895093:- | 94 |
| hsa-chr4_12502-3p | uugagaauuaugaugugccu | chr4:156810107-156810127:- | 52 |
| hsa-chr5_12656-3p | gcggcggcggcggugggcg | chr5:473425-473444:+ | 6218 |
| hsa-chr5_12656-5p | cucgcggucgcuguagccg | chr5:473378-473397:+ | 195 |
| hsa-chr5_12935-3p | uaacugguugaacaacuguaa | chr5:58999494-58999515:+ | 404 |
| hsa-chr5_12935-5p | guucaguuguucaaccaguuac | chr5:58999457-58999479:+ | 34 |
| hsa-chr5_13003-3p | uagggagcgggcgggcgg | chr5:72144675-72144693:+ | 626 |
| hsa-chr5_13504-3p | uggggagcgggaauggauaca | chr5:141229596-141229617:+ | 1 |
| hsa-chr5_13504-5p | uauccaguuccgcaauucacacu | chr5:141229560-141229583:+ | 41 |
| hsa-chr5_13505-3p | uagaugaggaagugaagcu | chr5:141296271-141296290:+ | 184 |
| hsa-chr5_13505-5p | uuugaguucuuucugugu | chr5:141296208-141296226:+ | 1 |
| hsa-chr5_13516-3p | uagcacagaauaguucaguug | chr5:142306605-142306626:+ | 8 |
| hsa-chr5_13516-5p | aauugaacugcucuaugcuacu | chr5:142306557-142306579:+ | 65 |
| *hsa-chr5_13669-3p* | *ucaccuggcauaagcaauucaca* | *chr5:167592904-167592927:+* | *351* |
| hsa-chr5_13669-5p | agaguugcuuaugucaggugaga | chr5:167592860-167592883:+ | 389 |
| hsa-chr5_13889-3p | ucauggaauccucagucgaau | chr5:204465-204486:- | 1 |
| hsa-chr5_13889-5p | aauugagccuguauaccuguu | chr5:204489-204510:- | 134 |
| hsa-chr5_13908-3p | acacacagagccaggccug | chr5:1102834-1102853:- | 252 |
| hsa-chr5_14426-3p | uuccugaaagaccugaag | chr5:90669930-90669948:- | 187 |
| hsa-chr5_14426-5p | auuugguagcagaaccuc | chr5:90669998-90670016:- | 1 |
| hsa-chr5_14666-3p | cuugacugaagcugauga | chr5:133380905-133380923:- | 74 |
| hsa-chr5_14766-3p | ccucuccacucccagccccgga | chr5:137799932-137799954:- | 61 |
| hsa-chr5_14766-5p | cgggcugggcgcgaagagagga | chr5:137799990-137800012:- | 18 |
| hsa-chr5_14985-3p | ucucaccugacauaagcaacucu | chr5:167592859-167592882:- | 26 |
| hsa-chr5_14985-5p | auugcuuaugccaggugagaga | chr5:167592899-167592921:- | 94 |
| hsa-chr5_15057-3p | aggggcaggggcagggagga | chr5:176307599-176307619:- | 2 |
| hsa-chr5_15057-5p | cuguacagccugggacuc | chr5:176307669-176307687:- | 37 |
| hsa-chr5_15070-3p | cugccaucuggugccagccuuu | chr5:176941854-176941876:- | 195 |
| hsa-chr5_15070-5p | uaggcuggccuggagggc | chr5:176941892-176941910:- | 11 |
| hsa-chr6_15182-3p | gacaauauuucuugccugguuu | chr6:2854312-2854334:+ | 3 |
| hsa-chr6_15182-5p | accaggcaagaacuacugucu | chr6:2854275-2854296:+ | 1044 |
| hsa-chr6_15289-3p | cggacuccugccucacuc | chr6:16592635-16592653:+ | 208 |
| hsa-chr6_15289-5p | aaaggugagggccuggcu | chr6:16592587-16592605:+ | 1 |
| hsa-chr6_15785-3p | uacuugaccuugacucucccuca | chr6:33169495-33169518:+ | 439 |
| hsa-chr6_15785-5p | ugggagagagaagggcugguucu | chr6:33169450-33169473:+ | 25 |
| hsa-chr6_16032-3p | cccucccuuuucuucuuuu | chr6:51833181-51833200:+ | 1 |
| hsa-chr6_16032-5p | uaggagaauggguaggcc | chr6:51833125-51833143:+ | 75 |
| hsa-chr6_17186-3p | ucgagaauugcguuuggacaau | chr6:33175633-33175655:- | 756 |
| hsa-chr6_17186-5p | acguccagacucaacucucggc | chr6:33175668-33175690:- | 86 |
| hsa-chr6_17545-3p | augauuucuccugugucccauagg | chr6:105821452-105821476:- | 1 |
| hsa-chr6_17545-5p | aaggaccguggaguuguguucc | chr6:105821490-105821512:- | 51 |
| hsa-chr6_17580-3p | cucgaggauugccagggc | chr6:109416010-109416028:- | 405 |
| hsa-chr6_17580-5p | gcuuugcgccucgggccc | chr6:109416062-109416080:- | 1 |
| hsa-chr6_17779-3p | gcuccuggccgcuggcug | chr6:147524542-147524560:- | 2 |
| hsa-chr6_17779-5p | cucgccguuaauggggug | chr6:147524563-147524581:- | 37 |
| hsa-chr6_17846-3p | ugagguaguagguggugugc | chr6:158914875-158914895:- | 450 |
| hsa-chr7_18283-3p | uuggcagagagcucuuaguaac | chr7:41738835-41738857:+ | 65 |
| hsa-chr7_18283-5p | aucugagaguacuuugucaagg | chr7:41738804-41738826:+ | 13 |
| hsa-chr7_19075-3p | cggcucuggccuccugacccaga | chr7:114562216-114562239:+ | 85 |
| hsa-chr7_19096-3p | accuuagaagucuaccuga | chr7:119435621-119435640:+ | 3 |
| hsa-chr7_19096-5p | ugggugggccuaggggug | chr7:119435573-119435591:+ | 29 |
| hsa-chr7_19202-3p | cacauccaucauuugccuucuucc | chr7:133352075-133352099:+ | 1 |
| *hsa-chr7_19202-5p* | *agcaaugaugaugacugaca* | *chr7:133352053-133352073:+* | *3470* |
| hsa-chr7_19400-3p | uugcacgcccaccgcccgaaa | chr7:155437238-155437259:+ | 3 |
| hsa-chr7_19400-5p | agccgggcggaggugacugccgc | chr7:155437206-155437229:+ | 123 |
| hsa-chr7_19480-3p | ucuguacccucaccucacccag | chr7:1538189-1538211:- | 526 |
| hsa-chr7_19480-5p | caggcggagugggggcacaggc | chr7:1538226-1538248:- | 3 |
| hsa-chr7_19496-3p | agccacugaugagccucugaggu | chr7:2815383-2815406:- | 379 |
| hsa-chr7_19496-5p | uuaggugauucugagccu | chr7:2815419-2815437:- | 5 |
| hsa-chr7_19686-3p | uaauagucaacuacuagccagu | chr7:27574619-27574641:- | 47 |
| hsa-chr7_19686-5p | cuggcuaguuguugacuauu | chr7:27574657-27574677:- | 16 |
| hsa-chr7_19740-3p | gauaccacuucugacacca | chr7:39592711-39592730:- | 49586 |
| hsa-chr7_19740-5p | gggggcaggggggagggg | chr7:39592773-39592791:- | 1 |
| hsa-chr7_20093-3p | aucauuuaugcuugcggaggac | chr7:92311783-92311805:- | 24 |
| hsa-chr7_20093-5p | gaccguaaggauacaaugauug | chr7:92311826-92311848:- | 48 |
| hsa-chr7_20361-3p | aaggacagccugagcccu | chr7:120369527-120369545:- | 1 |
| hsa-chr7_20361-5p | caguccaggcuguccugc | chr7:120369557-120369575:- | 44 |
| hsa-chr7_20363-3p | cucucucccccgcccccu | chr7:120496891-120496909:- | 1562 |
| hsa-chr7_20363-5p | gagggcggguggaggagg | chr7:120496953-120496971:- | 106 |
| hsa-chr7_20539-3p | cacaguguggcacagucguguc | chr7:142157354-142157376:- | 1 |
| hsa-chr7_20539-5p | ucggcuguguaucucugugcc | chr7:142157387-142157408:- | 84 |
| hsa-chr7_20648-3p | accagagggcucuagggc | chr7:158891322-158891340:- | 308 |
| hsa-chr8_20656-3p | gugggccgggcggggcua | chr8:494864-494882:+ | 1 |
| **hsa-chr8_20656-5p** | **gucccucccggccgccgg** | **chr8:494810-494828:+** | **1396** |
| hsa-chr8_20681-3p | cacaugagugcuuagaacaca | chr8:3961840-3961861:+ | 111 |
| hsa-chr8_20681-5p | aguucuaagcacccaugugca | chr8:3961804-3961825:+ | 14 |
| hsa-chr8_21031-3p | uuuuggauacuagcaggacgc | chr8:70560216-70560237:+ | 41 |
| hsa-chr8_21031-5p | uccugcuaguaucaaaaagcca | chr8:70560181-70560203:+ | 1 |
| hsa-chr8_21057-3p | auaucugcugaauucugcuga | chr8:75585012-75585033:+ | 126 |
| hsa-chr8_21057-5p | ugcugaauucuguugauauua | chr8:75584969-75584990:+ | 20 |
| hsa-chr8_21088-3p | cuguguggaggaucggugu | chr8:81398628-81398647:+ | 2 |
| hsa-chr8_21088-5p | cuccucggcgcgcggagg | chr8:81398602-81398620:+ | 2778 |
| hsa-chr8_21162-3p | cauggaagcacacuccuagca | chr8:97371013-97371034:+ | 1 |
| hsa-chr8_21162-5p | acuaggauugugcuucccugg | chr8:97370964-97370985:+ | 53 |
| hsa-chr8_21170-3p | uuuuuaaaaaguggcugu | chr8:98784662-98784680:+ | 1 |
| hsa-chr8_21170-5p | uugucacuacugcacuugacuagua | chr8:98784625-98784650:+ | 144521 |
| hsa-chr8_21303-3p | cucaucgaggugacuggccuugc | chr8:123965164-123965187:+ | 42 |
| hsa-chr8_21303-5p | aggccagcuuucuccagag | chr8:123965114-123965133:+ | 1 |
| hsa-chr8_21431-3p | gcccgccuggcccugggaa | chr8:143295534-143295553:+ | 71 |
| hsa-chr8_21657-3p | guucacauauaaagaagugacu | chr8:28924988-28925010:- | 7 |
| hsa-chr8_21657-5p | acagcuucucuauguguggauu | chr8:28925026-28925048:- | 107 |
| hsa-chr8_21781-3p | cuccacuguucuuggguc | chr8:53315266-53315284:- | 1 |
| hsa-chr8_21781-5p | gagcccaagcaguaacug | chr8:53315286-53315304:- | 50 |
| *hsa-chr8_21912-3p* | *auuucugggcuguagugcgcu* | *chr8:70835187-70835208:-* | *175166* |
| hsa-chr8_21912-5p | ugcaccuguggucccagcu | chr8:70835247-70835266:- | 13 |
| hsa-chr8_22064-3p | uuauccuccaguagacuaggga | chr8:99405894-99405916:- | 2918 |
| hsa-chr8_22064-5p | cccagccuacuggaggauaaga | chr8:99405931-99405953:- | 28 |
| hsa-chr8_22327-3p | aagugguggcccugaggc | chr8:140751013-140751031:- | 1 |
| *hsa-chr8_22327-5p* | *ccaggaccagcucugcgcccaggc* | *chr8:140751054-140751078:-* | *120* |
| *hsa-chr8_22338-3p* | *cauggcacuggaguagagcau* | *chr8:141181867-141181888:-* | *301* |
| hsa-chr8_22338-5p | cuccuaccccagaguccugcuu | chr8:141181916-141181938:- | 8 |
| hsa-chr8_22488-3p | uccgccgcagugcucuuggc | chr8:145158588-145158608:- | 1 |
| hsa-chr8_22488-5p | ggcggggcggcggcggcggc | chr8:145158621-145158641:- | 15664 |
| hsa-chr9_22799-3p | ucuucaggaacucuggcuaacu | chr9:37804261-37804283:+ | 82 |
| hsa-chr9_22799-5p | uuagccagaguuccuaaa | chr9:37804227-37804245:+ | 1 |
| hsa-chr9_22918-3p | ggacccgcuccucagggcuga | chr9:86535986-86536007:+ | 11 |
| *hsa-chr9_22918-5p* | *ugcgcucugaaaggccgggucccg* | *chr9:86535937-86535961:+* | *255* |
| hsa-chr9_22957-3p | cacccagcgcagugacug | chr9:91933765-91933783:+ | 509 |
| hsa-chr9_22957-5p | gucagcggcuacagaccc | chr9:91933712-91933730:+ | 2 |
| hsa-chr9_23127-3p | caggcagauaugugauaggcau | chr9:112273799-112273821:+ | 92 |
| hsa-chr9_23127-5p | ugccuaucaaauaucuaccug | chr9:112273761-112273782:+ | 33 |
| hsa-chr9_23251-3p | ccuccuguaacgggcucuggcu | chr9:129202453-129202475:+ | 35 |
| hsa-chr9_23251-5p | gcgagccccuugcuguauggagc | chr9:129202413-129202436:+ | 7 |
| hsa-chr9_23353-3p | agccugucugagcgccgcu | chr9:133282156-133282175:+ | 350911 |
| hsa-chr9_23671-3p | cucuacaguggucagcuuuuag | chr9:32456300-32456322:- | 2 |
| hsa-chr9_23671-5p | aaaagcuguccacuguagaguu | chr9:32456346-32456368:- | 534 |
| hsa-chr10_24598-3p | uuccccucccucgcugcc | chr10:21823202-21823220:+ | 1 |
| hsa-chr10_24598-5p | gacgcuccgaggaggaag | chr10:21823174-21823192:+ | 30 |
| hsa-chr10_24674-3p | uggguuggaguuagcucaagcgg | chr10:31840056-31840079:+ | 721 |
| *hsa-chr10_24674-5p* | *cgcgggugcuuacugacccu* | *chr10:31840034-31840054:+* | *7642* |
| hsa-chr10_24736-3p | cguguccagaauggccagccaga | chr10:43966722-43966745:+ | 5 |
| hsa-chr10_24736-5p | uggcuggcugcuccgggcacu | chr10:43966686-43966707:+ | 415 |
| hsa-chr10_24907-3p | caucugauggggaauggccugc | chr10:72082714-72082736:+ | 58 |
| hsa-chr10_24907-5p | aggccauuccccaucagauggg | chr10:72082675-72082697:+ | 6 |
| hsa-chr10_25108-3p | ccccccugguuugcaggccuuu | chr10:94624544-94624566:+ | 1 |
| hsa-chr10_25108-5p | ucaggucuucagacuguggggc | chr10:94624510-94624532:+ | 180 |
| hsa-chr10_25195-3p | cauugucuuucugucucucca | chr10:102798376-102798397:+ | 17 |
| hsa-chr10_25216-5p | ugcggcggcuucagcucaggcc | chr10:104210199-104210221:+ | 89 |
| **hsa-chr10_25333-3p** | **ugagcucucugcacucccaggc** | **chr10:121201807-121201829:+** | **182** |
| hsa-chr10_25333-5p | ggggagcacaggggccccaga | chr10:121201772-121201793:+ | 1 |
| hsa-chr10_25470-5p | uuagggcccuggcuccauc | chr10:135055629-135055648:+ | 1325 |
| hsa-chr10_25556-3p | uauauaguauaugugcauguau | chr10:9361012-9361034:- | 77 |
| hsa-chr10_25556-5p | augugcacauauauuauauaug | chr10:9361051-9361073:- | 1 |
| hsa-chr10_25646-3p | uggcggcggcggcgggggcggcggg | chr10:27541493-27541518:- | 53752 |
| hsa-chr10_25646-5p | ccgcccccacacacgcaca | chr10:27541542-27541561:- | 1 |
| hsa-chr10_25658-3p | ucacugacgguuucugcuugccu | chr10:29783931-29783954:- | 39 |
| hsa-chr10_25658-5p | ccaagcaguuucugaucagaaga | chr10:29783972-29783995:- | 2 |
| hsa-chr10_25936-3p | uccuucugugcugugguccagg | chr10:79566685-79566707:- | 38 |
| hsa-chr10_26078-3p | ucuggcugcuauggcccccucc | chr10:99635579-99635601:- | 94 |
| hsa-chr10_26078-5p | aggugccauucugagggccaggagu | chr10:99635616-99635641:- | 14 |
| hsa-chr10_26308-3p | uccuccugcccuccuugcug | chr10:126784000-126784020:- | 21 |
| hsa-chr10_26308-5p | accaacggagcuguucccagguuca | chr10:126784051-126784076:- | 1 |
| hsa-chr11_26539-3p | cgccguccuccccccucccc | chr11:1357555-1357575:+ | 5080 |
| hsa-chr11_26539-5p | gccagggaccuggggcuu | chr11:1357504-1357522:+ | 1 |
| hsa-chr11_26572-3p | agcacaaaguuucugagcgccu | chr11:3863030-3863052:+ | 42 |
| hsa-chr11_26572-5p | ugcgcucggagcuuugacuuga | chr11:3862989-3863011:+ | 87 |
| hsa-chr11_26648-3p | aagcuucugcaucuuuucuuccc | chr11:12343097-12343120:+ | 5 |
| hsa-chr11_26648-5p | gcaggaagagacgcagcagcuug | chr11:12343060-12343083:+ | 42 |
| hsa-chr11_27009-3p | ccugaacuugcggaaccc | chr11:62573084-62573102:+ | 179 |
| hsa-chr11_27009-5p | gccgucguucgaggggcgg | chr11:62573037-62573056:+ | 1 |
| hsa-chr11_27039-3p | ugcugauccucucccaccccaga | chr11:64024068-64024091:+ | 107 |
| hsa-chr11_27039-5p | uggggugcagguggguggg | chr11:64024025-64024044:+ | 3 |
| hsa-chr11_27174-3p | ccuguugggacagucaguugguau | chr11:66394633-66394657:+ | 31 |
| hsa-chr11_27174-5p | uacccugacugucccucuguag | chr11:66394594-66394616:+ | 1241 |
| hsa-chr11_27445-3p | auucgcugggaauucagccucu | chr11:100841641-100841663:+ | 247 |
| hsa-chr11_27445-5p | gaggcugaauucccagugagug | chr11:100841606-100841628:+ | 18 |
| *hsa-chr11_27716-3p* | *cugcccaucccaccccagcaucc* | *chr11:418400-418423:-* | *512* |
| hsa-chr11_27716-5p | aggcuggguggggugggggcaggc | chr11:418439-418463:- | 9 |
| hsa-chr11_28374-3p | caucugugaccccaccucuag | chr11:64568503-64568524:- | 21 |
| hsa-chr11_28374-5p | gagggguaggggccacagagcagg | chr11:64568538-64568562:- | 3 |
| hsa-chr11_28952-3p | ugggggcggcggcggggga | chr11:133825798-133825817:- | 3556 |
| hsa-chr11_28952-5p | ccccacgccgcccucccc | chr11:133825838-133825856:- | 1 |
| hsa-chr12_29012-3p | caggggaggccugggauucugu | chr12:4834671-4834693:+ | 18 |
| hsa-chr12_29012-5p | agauucucaggcucuaccccaga | chr12:4834632-4834655:+ | 111 |
| hsa-chr12_29077-3p | ucugacccucugcuucccccagg | chr12:6965447-6965470:+ | 129 |
| hsa-chr12_29137-3p | augcaugcugggcuuguaaccu | chr12:11403694-11403716:+ | 106 |
| hsa-chr12_29137-5p | uuacaagcccaguaugcauuagg | chr12:11403660-11403683:+ | 2 |
| hsa-chr12_29168-3p | agugccuccuugaaaucugugc | chr12:13041642-13041664:+ | 1 |
| hsa-chr12_29168-5p | ucaggauuuaaaggggcacuc | chr12:13041605-13041626:+ | 153 |
| hsa-chr12_29295-3p | caaaaccugcaguuacuuuugc | chr12:45513646-45513668:+ | 1286 |
| hsa-chr12_29295-5p | aaaaguaacugcaggguuugcc | chr12:45513595-45513617:+ | 1 |
| hsa-chr12_30063-3p | acccuggaggcuggugagg | chr12:127613590-127613609:+ | 28 |
| hsa-chr12_30159-3p | gcaccaccuggcggggag | chr12:5950236-5950254:- | 5 |
| hsa-chr12_30159-5p | cuggcucagcgugugccu | chr12:5950269-5950287:- | 151 |
| hsa-chr12_30166-3p | cucaccggcccgcguccccgcagc | chr12:6438787-6438811:- | 268 |
| hsa-chr12_30166-5p | gggcgcgggaugcggggc | chr12:6438835-6438853:- | 3 |
| hsa-chr12_30306-3p | ucccuuuucugguaguucuca | chr12:26957042-26957063:- | 141 |
| hsa-chr12_30306-5p | aggaauuaccugggaaugggaagu | chr12:26957082-26957106:- | 3 |
| hsa-chr12_30550-3p | uugcaaaggaauccugggccu | chr12:56360207-56360228:- | 67 |
| hsa-chr12_30648-3p | ucccagcuggucauuaauccuc | chr12:66251817-66251839:- | 36 |
| hsa-chr12_30648-5p | gaauuaauggcuggcugggag | chr12:66251863-66251884:- | 13 |
| hsa-chr12_30711-3p | ugauuagacaggagccagacac | chr12:81300350-81300372:- | 1 |
| hsa-chr12_30711-5p | ucuggcuccuuucuaaucacu | chr12:81300389-81300410:- | 119 |
| hsa-chr12_30729-5p | uuuccucucugccccauagggug | chr12:87138890-87138913:- | 180 |
| hsa-chr12_30797-3p | gaucugcgaagcccccucccca | chr12:98909617-98909639:- | 1 |
| hsa-chr12_30797-5p | accgaggggucuuccaggaacucc | chr12:98909650-98909674:- | 83 |
| hsa-chr12_30850-3p | uaggucacuggggucagagc | chr12:108141103-108141123:- | 54 |
| hsa-chr12_30850-5p | ucugacgccaguguccuauguuc | chr12:108141129-108141152:- | 2 |
| hsa-chr13_31211-3p | uaugugccuaguggcugcugucu | chr13:27259514-27259537:+ | 441 |
| hsa-chr13_31211-5p | agaguagccacuagccacaugu | chr13:27259471-27259493:+ | 59 |
| hsa-chr13_31264-5p | gcggccuguccccagugc | chr13:36104475-36104493:+ | 2034 |
| hsa-chr13_31272-3p | cucgguggcggccgcggcc | chr13:37393599-37393618:+ | 7 |
| hsa-chr13_31272-5p | ccggcugcggcucccaccuugg | chr13:37393570-37393592:+ | 100 |
| hsa-chr13_31406-3p | caguauguucuggucccucugg | chr13:55195341-55195363:+ | 7 |
| hsa-chr13_31406-5p | uagagggaccagaacauacugu | chr13:55195300-55195322:+ | 118 |
| hsa-chr13_31480-3p | ugugcaggcuugaggauuaggu | chr13:76353310-76353332:+ | 12 |
| hsa-chr13_31480-5p | uuaauccucaagccuguacaacc | chr13:76353266-76353289:+ | 337 |
| hsa-chr13_31665-3p | cagcucaggcuuggaaug | chr13:112069950-112069968:+ | 35 |
| *hsa-chr13_31956-3p* | *uuguuuucaacuuacaaugggu* | *chr13:48602648-48602670:-* | *71* |
| hsa-chr13_31956-5p | ucguuguaaguugaaaauauag | chr13:48602684-48602706:- | 27 |
| hsa-chr13_31997-3p | caguauguucuggucccucu | chr13:55195300-55195320:- | 2 |
| *hsa-chr13_31997-5p* | *agagggaccagaacauacugugg* | *chr13:55195337-55195360:-* | *66* |
| hsa-chr13_32015-3p | agacauguuucuugcauacugu | chr13:60538557-60538579:- | 12 |
| hsa-chr13_32015-5p | acaguauacaaaaaacaugucug | chr13:60538597-60538620:- | 37 |
| hsa-chr13_32223-3p | cccuccucgccgcucccc | chr13:112720935-112720953:- | 2 |
| hsa-chr13_32223-5p | gcgcgcggcggcggcggcggcggc | chr13:112720967-112720991:- | 222182 |
| hsa-chr13_32229-3p | ucggggucggggucgggccc | chr13:114144927-114144947:- | 6 |
| hsa-chr13_32229-5p | uggcccggcgucgccucugc | chr13:114144956-114144976:- | 138 |
| hsa-chr14_32275-3p | ucugagcccuguucucccuagg | chr14:21460230-21460252:+ | 228 |
| hsa-chr14_32589-3p | agcaaggcggcaucucucuccu | chr14:65801877-65801899:+ | 1413 |
| hsa-chr14_32589-5p | ucagagagaugccgccuugcu | chr14:65801842-65801863:+ | 84 |
| hsa-chr14_32682-3p | aaaaaccgugauuacuuuugca | chr14:75899986-75900008:+ | 2692 |
| hsa-chr14_32682-5p | aaaaguaaucgcggguuuugcc | chr14:75899949-75899971:+ | 207 |
| hsa-chr14_32791-3p | uuuugugugucagggugcaggu | chr14:94580022-94580044:+ | 322 |
| hsa-chr14_32791-5p | aggccuccagacacaccgcagc | chr14:94579986-94580008:+ | 12 |
| hsa-chr14_33061-3p | gccuguggcuguggcugc | chr14:103754711-103754729:+ | 29 |
| hsa-chr14_33061-5p | cccacugaccccaggcuc | chr14:103754687-103754705:+ | 1 |
| hsa-chr14_33099-3p | uggggcugugucacugugggu | chr14:106373051-106373072:+ | 143 |
| hsa-chr14_33160-3p | agguccgcagcugcucugccu | chr14:23447393-23447414:- | 42 |
| hsa-chr14_33160-5p | acagagcagcuguuggauccc | chr14:23447428-23447449:- | 13 |
| hsa-chr14_33230-3p | cuugagaaucggaaggcccagc | chr14:32434762-32434784:- | 77 |
| hsa-chr14_33230-5p | gaugggccuuccgacucccaaggc | chr14:32434802-32434826:- | 3 |
| hsa-chr14_33646-3p | uugcucugcucucccuuguacu | chr14:96000143-96000165:- | 531 |
| hsa-chr14_33646-5p | ugcagaggagacaaagcaagccu | chr14:96000180-96000203:- | 2 |
| hsa-chr14_33679-3p | acuccuguaugaagccguuccc | chr14:101340849-101340871:- | 31 |
| hsa-chr14_33679-5p | agaaaggcaucauauaggagcug | chr14:101340886-101340909:- | 60 |
| hsa-chr15_33850-3p | cucacagugacacaagcccccac | chr15:20213684-20213707:+ | 6 |
| hsa-chr15_33850-5p | uggggcugugucacuguggg | chr15:20213637-20213657:+ | 110 |
| hsa-chr15_34132-3p | guccccccacuccccggcagg | chr15:45409269-45409290:+ | 2 |
| hsa-chr15_34132-5p | accccggugggcugggagaagcc | chr15:45409234-45409257:+ | 222 |
| hsa-chr15_34456-3p | agcaaugugugauuaggaaaagaa | chr15:85697801-85697825:+ | 2 |
| hsa-chr15_34456-5p | uuuccuaaucacauauugcuuc | chr15:85697762-85697784:+ | 59 |
| hsa-chr15_34527-3p | caggaacuggccgggguc | chr15:91427704-91427722:+ | 42 |
| hsa-chr15_34527-5p | cugggccaacugaaaccg | chr15:91427665-91427683:+ | 1 |
| hsa-chr15_34709-3p | gcuggcgggggcgguggc | chr15:40733594-40733612:- | 3 |
| hsa-chr15_34709-5p | ucguccguccguccgucc | chr15:40733620-40733638:- | 23101 |
| hsa-chr15_34840-3p | gcggcggcggcggcggcggcg | chr15:56535983-56536004:- | 118246 |
| hsa-chr15_34840-5p | ggccgcggcggcggcggg | chr15:56536015-56536033:- | 6220 |
| hsa-chr15_34842-3p | cucggccuuugcucgcagcacu | chr15:56657482-56657504:- | 396 |
| hsa-chr15_34842-5p | gugcugcgagcggcgcgagccucu | chr15:56657513-56657537:- | 2 |
| hsa-chr16_35593-3p | ucugaggagcgcggggcgcggccauga | chr16:18995363-18995390:+ | 1 |
| hsa-chr16_35593-5p | gcggcggcggcggcggcg | chr16:18995322-18995340:+ | 103536 |
| hsa-chr16_35703-3p | ugcuagaccucacagggccaga | chr16:28858356-28858378:+ | 32 |
| hsa-chr16_35703-5p | ugggcccugugaggucaggcagc | chr16:28858316-28858339:+ | 4 |
| hsa-chr16_35900-3p | cugccgcugcucccaagcc | chr16:48643945-48643964:+ | 1 |
| hsa-chr16_35900-5p | ccgggggccggcggcggcg | chr16:48643910-48643929:+ | 3278 |
| hsa-chr16_35996-3p | ugagggagcaguggcuggggug | chr16:57673406-57673428:+ | 2 |
| *hsa-chr16_35996-5p* | *ucucagccucuauucccuggc* | *chr16:57673361-57673382:+* | *121* |
| hsa-chr16_36010-3p | cugcgggccugagcgccguu | chr16:58061460-58061480:+ | 30 |
| hsa-chr16_36010-5p | aacggugcucaggccuuggugc | chr16:58061422-58061444:+ | 207 |
| hsa-chr16_36049-3p | cugacugcccuggccuggccagg | chr16:67469509-67469532:+ | 354 |
| hsa-chr16_36049-5p | aggcuaggcugggccacagugg | chr16:67469480-67469502:+ | 3 |
| hsa-chr16_36196-3p | cgaaggcuggucagggagaca | chr16:81511774-81511795:+ | 16 |
| hsa-chr16_36196-5p | uaucccagggccagucuccacu | chr16:81511737-81511759:+ | 80 |
| hsa-chr16_36327-3p | uccugccgccaggagcucacc | chr16:89766811-89766832:+ | 34 |
| hsa-chr16_36327-5p | agagcucccggcggcccugc | chr16:89766787-89766807:+ | 11 |
| hsa-chr16_36443-5p | cgcgcggggggcccgggc | chr16:2827158-2827176:- | 29864 |
| hsa-chr16_36566-3p | uuucugcgaccaaucagacu | chr16:15669649-15669669:- | 342 |
| hsa-chr16_36566-5p | aucugauuggucgcagaaagc | chr16:15669681-15669702:- | 47 |
| hsa-chr16_36639-3p | caggcgcuccacggcggc | chr16:22201693-22201711:- | 1 |
| hsa-chr16_36639-5p | cgggcgugggcgagggug | chr16:22201753-22201771:- | 31 |
| hsa-chr16_36860-3p | augaguacgggaucucuguaga | chr16:53644724-53644746:- | 55 |
| hsa-chr16_36860-5p | aucgggguucucugacucacu | chr16:53644758-53644779:- | 15 |
| hsa-chr16_37169-3p | ggugagggugucugagccca | chr16:88797727-88797747:- | 10 |
| hsa-chr16_37169-5p | ucggccgugaugccucacaccca | chr16:88797774-88797797:- | 70 |
| hsa-chr17_37536-3p | aucagagauggaaauuauuauc | chr17:20841758-20841780:+ | 2 |
| hsa-chr17_37536-5p | auaauagucuccaacucugauu | chr17:20841721-20841743:+ | 44 |
| hsa-chr17_37663-3p | acauugacugacuuuugagu | chr17:29060894-29060914:+ | 84 |
| hsa-chr17_37663-5p | uuuuucugcaguuuguguccgaga | chr17:29060861-29060885:+ | 2 |
| hsa-chr17_37693-3p | ucccuguccuccaggagcu | chr17:31555904-31555923:+ | 3204 |
| hsa-chr17_37789-3p | cuuccuccuggcugggccugccc | chr17:37831468-37831491:+ | 84 |
| hsa-chr17_37789-5p | acaggcucccaggaggagggu | chr17:37831434-37831455:+ | 34 |
| hsa-chr17_38046-5p | ccaggggagcccggcggg | chr17:56395786-56395804:+ | 8037 |
| hsa-chr17_38208-3p | cuggaguaggagggcagcc | chr17:69867613-69867632:+ | 1 |
| *hsa-chr17_38208-5p* | *guuguauuucaacuaaaaa* | *chr17:69867569-69867588:+* | *345* |
| hsa-chr17_38267-3p | accccuccccacuccucgccagg | chr17:73513068-73513091:+ | 42 |
| hsa-chr17_38267-5p | ggggcgaggcgggccgcgggguc | chr17:73513025-73513048:+ | 1 |
| *hsa-chr17_38309-3p* | *gaagcagcgccugucgcaacucgcc* | *chr17:76136873-76136898:+* | *1054* |
| hsa-chr17_38309-5p | agaguugcugccgcugcuguccu | chr17:76136835-76136858:+ | 130 |
| hsa-chr17_38565-3p | gaggccauucggcucugaggucc | chr17:7210149-7210172:- | 78 |
| hsa-chr17_38565-5p | acccucaguccguauuggucuc | chr17:7210185-7210207:- | 314 |
| hsa-chr17_38641-3p | gcuccugggacuggcucugca | chr17:15466696-15466717:- | 3 |
| hsa-chr17_38641-5p | agggcuggcguagggccgc | chr17:15466763-15466782:- | 35 |
| hsa-chr17_38865-3p | ggguucaaauccaggcucug | chr17:36702549-36702569:- | 1 |
| hsa-chr17_38865-5p | acuggccuuggagucaga | chr17:36702575-36702593:- | 4939 |
| hsa-chr17_38898-3p | aggccggagucuaagggc | chr17:37793253-37793271:- | 117 |
| hsa-chr17_38898-5p | cuccagccccggcccugc | chr17:37793287-37793305:- | 1 |
| hsa-chr17_39086-3p | cggcggccgccuggggug | chr17:44450029-44450047:- | 75 |
| hsa-chr17_39086-5p | gucccuggccgcgucccc | chr17:44450081-44450099:- | 1 |
| hsa-chr17_39090-3p | cggcggccgccuggggug | chr17:44667950-44667968:- | 75 |
| hsa-chr17_39090-5p | gucccuggccgcgucccc | chr17:44668002-44668020:- | 1 |
| hsa-chr17_39116-3p | ccccugggcuguuacuguucc | chr17:46199546-46199567:- | 329 |
| hsa-chr17_39116-5p | agacaguaacagcccgggacagcc | chr17:46199583-46199607:- | 182 |
| hsa-chr17_39137-3p | cccgacagggagguggccggg | chr17:46719966-46719987:- | 102 |
| hsa-chr17_39137-5p | gcgacugccucccugcugugagc | chr17:46720005-46720028:- | 672 |
| hsa-chr17_39372-3p | ggggggccggcggcggcggcggc | chr17:65822070-65822093:- | 40600 |
| hsa-chr17_39372-5p | cgcccccgccuccucgcc | chr17:65822120-65822138:- | 1 |
| hsa-chr18_40060-5p | gaggagagagcgagagug | chr18:72922808-72922826:+ | 87 |
| hsa-chr18_40201-3p | ggcagagguuuucuggaaccagc | chr18:21141858-21141881:- | 2 |
| hsa-chr18_40201-5p | aaguuccauaauucucuggcu | chr18:21141895-21141916:- | 96 |
| hsa-chr18_40274-3p | auugaggagccugaagau | chr18:37645901-37645919:- | 46 |
| hsa-chr18_40274-5p | uuuuuguuuguuuguuuguuu | chr18:37645940-37645961:- | 80 |
| hsa-chr19_40551-3p | uugccucucccgucccuguagu | chr19:3201500-3201522:+ | 666 |
| hsa-chr19_40551-5p | cauggggugcgggagccgggcgggggu | chr19:3201463-3201490:+ | 5 |
| hsa-chr19_40574-3p | acucugcucccuccccccaga | chr19:4215955-4215976:+ | 4 |
| hsa-chr19_40574-5p | cacgggggcugagagcagaacc | chr19:4215924-4215946:+ | 78 |
| hsa-chr19_40606-3p | cggggaaagggccgggaagggc | chr19:5978351-5978373:+ | 2 |
| hsa-chr19_40606-5p | caucucccgccuccuuuucccgcc | chr19:5978316-5978340:+ | 63 |
| hsa-chr19_40626-3p | cauggagcccccucuggguggacu | chr19:7573106-7573130:+ | 1 |
| hsa-chr19_40626-5p | cuguccacccugucuccacagu | chr19:7573071-7573093:+ | 85 |
| hsa-chr19_40801-3p | cuuccccacccucuccugcag | chr19:13063482-13063503:+ | 111 |
| hsa-chr19_40801-5p | uggggagaggaggggaccagggc | chr19:13063444-13063467:+ | 4 |
| hsa-chr19_40877-3p | ugggccuuucugucucugcagg | chr19:16198816-16198838:+ | 138 |
| hsa-chr19_40877-5p | ugcaguggaugggagaggacacggc | chr19:16198771-16198796:+ | 4 |
| hsa-chr19_40928-3p | agacagacgcagguacacacag | chr19:18395530-18395552:+ | 5 |
| hsa-chr19_40928-5p | gugugugcaccugugucugucu | chr19:18395493-18395515:+ | 248 |
| hsa-chr19_41088-3p | ucucauuggucaggccugaguc | chr19:35652467-35652489:+ | 24 |
| hsa-chr19_41088-5p | ucaugucugaaccaaugagagc | chr19:35652429-35652451:+ | 120 |
| hsa-chr19_41115-3p | gcggcggcggcgggcggcg | chr19:36208924-36208943:+ | 10972 |
| hsa-chr19_41115-5p | cccuccccccgccucccc | chr19:36208882-36208900:+ | 6 |
| hsa-chr19_41254-5p | ucugcacuacagaacuuuga | chr19:41049427-41049447:+ | 90 |
| hsa-chr19_41545-3p | ucuguccaccuugcuucuucagg | chr19:48981510-48981533:+ | 227 |
| hsa-chr19_41545-5p | ugagggaggugggguggggugaggac | chr19:48981470-48981496:+ | 1 |
| hsa-chr19_41672-3p | ugaugauagggccaugggccacc | chr19:52303236-52303259:+ | 8 |
| hsa-chr19_41672-5p | uagcccauggcucgauccucagc | chr19:52303197-52303220:+ | 236 |
| hsa-chr19_41683-3p | caccgcucucuccaccccgagu | chr19:52772982-52773004:+ | 54 |
| hsa-chr19_41683-5p | cccgggauguggggggcgguacaga | chr19:52772943-52772968:+ | 1 |
| hsa-chr19_41779-3p | ucugcucucucccacccgcagu | chr19:54651862-54651884:+ | 133 |
| hsa-chr19_41900-3p | ugaccugcacucucuccccagg | chr19:652295-652317:- | 181 |
| hsa-chr19_41900-5p | gaggggagggagcgccugg | chr19:652347-652366:- | 2 |
| hsa-chr19_42230-3p | cuucggcgcucuucccccaga | chr19:12788024-12788045:- | 243 |
| hsa-chr19_42230-5p | agaggggaaggagggcug | chr19:12788063-12788081:- | 2 |
| hsa-chr19_42744-3p | ugugccugggacuccacc | chr19:41195759-41195777:- | 79 |
| hsa-chr19_42748-3p | ugcacgcgaccauagagccuu | chr19:41769347-41769368:- | 700 |
| hsa-chr19_42748-5p | uugcucuauggucggguaccuga | chr19:41769384-41769407:- | 22 |
| hsa-chr19_42818-3p | uuuaaaguucuagaaccguga | chr19:44142007-44142028:- | 91 |
| hsa-chr19_42818-5p | aggauucuagaauccagaaggcu | chr19:44142044-44142067:- | 1 |
| hsa-chr19_43042-3p | cgcgcuccuccccugccccagc | chr19:55603911-55603933:- | 117 |
| hsa-chr19_43042-5p | ggcggggugggagagguggg | chr19:55603964-55603984:- | 1 |
| hsa-chr20_43152-3p | ucagccgcagccgaggccaag | chr20:3452031-3452052:+ | 1 |
| hsa-chr20_43152-5p | gcggcggcggcggcggcg | chr20:3452004-3452022:+ | 77320 |
| hsa-chr20_43329-3p | ucccuguucucuguguuugcagc | chr20:25262647-25262670:+ | 186 |
| hsa-chr20_43329-5p | uugcucaccuggugcagggc | chr20:25262610-25262630:+ | 6 |
| hsa-chr20_43415-3p | uaaauuaucugagccccaggaa | chr20:33627731-33627753:+ | 1388 |
| hsa-chr20_43415-5p | ccuggggcucagauaauuuaca | chr20:33627694-33627716:+ | 32 |
| hsa-chr20_43441-3p | guggcggcggcgggggugu | chr20:34681441-34681460:+ | 2889 |
| hsa-chr20_43441-5p | gguccaggcgccgccgcc | chr20:34681391-34681409:+ | 7 |
| hsa-chr20_43572-3p | uaagaguuguucuccauuucaga | chr20:43710020-43710043:+ | 2 |
| hsa-chr20_43572-5p | aagaauggagagacacuuguagu | chr20:43709976-43709999:+ | 48 |
| hsa-chr20_43828-3p | gcucugacuggaagcccc | chr20:61507189-61507207:+ | 1 |
| hsa-chr20_43828-5p | aggcuguagguccgagag | chr20:61507159-61507177:+ | 81 |
| hsa-chr20_44178-3p | uaaauuaucugagccccaggaa | chr20:33627691-33627713:- | 1388 |
| hsa-chr20_44178-5p | ccuggggcucagauaauuuaca | chr20:33627728-33627750:- | 32 |
| hsa-chr20_44187-5p | gcugggggugacuuagagcca | chr20:34194183-34194204:- | 40 |
| hsa-chr20_44288-3p | cggcccggcgcuaagcug | chr20:42142605-42142623:- | 265 |
| hsa-chr20_44288-5p | cugugcuggugcuggagcu | chr20:42142626-42142645:- | 1 |
| hsa-chr20_44334-3p | ucuuacccucccuccacuuccagu | chr20:44596272-44596296:- | 92 |
| hsa-chr20_44334-5p | uuggacugcauggguagggg | chr20:44596328-44596348:- | 1 |
| hsa-chr20_44340-3p | gugccugucccccgcccugcagg | chr20:44983603-44983626:- | 51 |
| hsa-chr20_44340-5p | gcagggccggaccagaccug | chr20:44983629-44983649:- | 3 |
| hsa-chr20_44415-3p | uguauguacacauaugccuaac | chr20:50733192-50733214:- | 1 |
| hsa-chr20_44415-5p | uugggcauauguguauauaugu | chr20:50733229-50733251:- | 111 |
| hsa-chr20_44428-3p | ccgggagucuuggggcgccu | chr20:52209640-52209660:- | 32 |
| hsa-chr20_44428-5p | cgcgccccgugacucccucgggug | chr20:52209675-52209699:- | 3 |
| hsa-chr21_44596-5p | ucucugggccugugucuu | chr21:10205174-10205192:+ | 156 |
| hsa-chr21_44759-3p | ucucccuuccugcccuggccu | chr21:43741458-43741479:+ | 43 |
| hsa-chr21_44946-3p | caaaaccugcaguuacuuuugc | chr21:33581807-33581829:- | 1286 |
| hsa-chr21_44946-5p | aaaaguuauugcaguuuuugcc | chr21:33581840-33581862:- | 57 |
| hsa-chr22_45125-3p | ugugcaggugcugugugggc | chr22:19945212-19945232:+ | 10 |
| hsa-chr22_45125-5p | cccucacacgugcaucugcaugu | chr22:19945170-19945193:+ | 1 |
| hsa-chr22_45145-3p | ucagacagggcuccccgcaccg | chr22:20136528-20136550:+ | 61 |
| hsa-chr22_45184-3p | uucgcgggggcgucggug | chr22:22222144-22222162:+ | 1 |
| hsa-chr22_45184-5p | cggcguugacugaggggg | chr22:22222091-22222109:+ | 80 |
| hsa-chr22_45253-3p | ccugccugugcucugggc | chr22:25782158-25782176:+ | 122 |
| hsa-chr22_45253-5p | cuggggcaggcggggagg | chr22:25782130-25782148:+ | 1 |
| hsa-chr22_45305-3p | cggaaccuuagagcuucagcca | chr22:31556086-31556108:+ | 100 |
| hsa-chr22_45305-5p | gccgaagcuccaagguuccuc | chr22:31556048-31556069:+ | 1 |
| hsa-chr22_45323-3p | acccuguccuccaggagcuc | chr22:32524336-32524356:+ | 162 |
| hsa-chr22_45348-3p | aaagaccgcgauuacuuuugca | chr22:36736259-36736281:+ | 44 |
| hsa-chr22_45348-5p | aaaaguaaucgcggucuuugcc | chr22:36736222-36736244:+ | 608 |
| hsa-chr22_45590-3p | ggaccuuggacauccacuuucu | chr22:50845920-50845942:+ | 1 |
| hsa-chr22_45590-5p | cagguggaggugugagguccugg | chr22:50845885-50845908:+ | 32 |
| hsa-chr22_45621-3p | ccccaggcccugcagagcug | chr22:18313790-18313810:- | 32 |
| hsa-chr22_45621-5p | ucccugcagcggucagaggauc | chr22:18313827-18313849:- | 72 |
| hsa-chr22_45692-3p | ucagacagggcuccccgcaccg | chr22:23736584-23736606:- | 61 |
| hsa-chr22_45835-3p | aaagaccgcgauuacuuuugca | chr22:36736218-36736240:- | 44 |
| hsa-chr22_45835-5p | aaaaguaaucgcggucuuugcc | chr22:36736255-36736277:- | 608 |
| hsa-chr22_45886-3p | guccggcugccgcgcauc | chr22:38668924-38668942:- | 1 |
| hsa-chr22_45886-5p | gccggcggcggcggagacu | chr22:38668976-38668995:- | 6101 |
| hsa-chrX_46131-3p | uuccgacaccaugacaug | chrX:3733127-3733145:+ | 77 |
| hsa-chrX_46131-5p | gucaaugccgucugaccc | chrX:3733086-3733104:+ | 1 |
| hsa-chrX_46172-3p | guacuaugagguucugcauuuc | chrX:14485483-14485505:+ | 3 |
| hsa-chrX_46172-5p | aaugcagccugaguaguacu | chrX:14485447-14485467:+ | 690 |
| hsa-chrX_46249-3p | uucagacuuaaaaaaggauacu | chrX:28036739-28036761:+ | 48 |
| hsa-chrX_46249-5p | uauccuuuucuagggcugaaca | chrX:28036702-28036724:+ | 4 |
| hsa-chrX_46323-3p | aggagauugugaagaaac | chrX:41535962-41535980:+ | 52 |
| hsa-chrX_46323-5p | uuaaaaggaucacucugg | chrX:41535934-41535952:+ | 7 |
| hsa-chrX_46436-3p | ugccucagguuccucagcuaga | chrX:55028362-55028384:+ | 3 |
| hsa-chrX_46436-5p | uagcugugcacccucuggcaag | chrX:55028324-55028346:+ | 110 |
| hsa-chrX_46437-3p | uccuguuuccucuggguc | chrX:55187721-55187739:+ | 157 |
| hsa-chrX_46437-5p | cccggagggcggggcugg | chrX:55187652-55187670:+ | 1 |
| hsa-chrX_46790-3p | gccgccgccgccgcugcug | chrX:129118155-129118174:+ | 80 |
| hsa-chrX_46790-5p | cggcgggcggcggggcgg | chrX:129118106-129118124:+ | 14403 |
| hsa-chrX_46983-3p | caggcucaagcgauccucc | chrX:3731599-3731618:- | 21 |
| hsa-chrX_46983-5p | agggaggaucgcuucagccugg | chrX:3731662-3731684:- | 872 |

**Supplementary Table S2. Patient and tumor characteristics of the 38 patients included in the comparison of primary tumors and metastases.**

| **Patient** | **Sex** | **Age** | **Tissue Type** | **Location** | **Morphology** | **Differentiation** | **Tumor cellularity (%)** | **MS status** | **T** | **N** | **M** | **Time to Resection (days)** | **Chemotherapy** | **Normal Tissue** | **Mucosa (%)** |
| --- | --- | --- | --- | --- | --- | --- | --- | --- | --- | --- | --- | --- | --- | --- | --- |
| 1 | M | 50 | P | Coecum | Tubular AC | Moderate | >70% | MSS | 3 | 2/13 | 0 |  |  | Yes | 60% |
|  |  |  | M | Lung | Tubular AC |  | >70% |  |  |  |  | 1372 | Ap, 6x 5-FU/LV | Yes |  |
| 2 | F | 63 | P | Sigmoid | Tubular AC | Moderate | >70% | MSS | 3 | 6/6 | 1 |  |  | Yes | 40% |
|  |  |  | M | Liver | Tubular AC |  | >70% |  |  |  |  | 77 | No | Yes |  |
| 3 | M | 69 | P | Coecum | Tubular AC | Moderate | 60% | MSS | 3 | 14/20 | 0 |  |  | No |  |
|  |  |  | M | Liver | Tubular AC |  | >70% |  |  |  |  | 1619 | Ap, 7x xelox | Yes |  |
| 4 | F | 71 | P | Transversum | Tubular AC | Moderate | >70% | MSS | 3 | 0/13 | 0 |  |  | Yes | 20% |
|  |  |  | M | Liver | Tubular AC |  | >70% |  |  |  |  | 757 | No | No |  |
| 5 | F | 43 | P | Coecum | Tubular AC | Moderate | >70% | MSI-low | 3 | 0/21 | 0 |  |  | Yes | 90% |
|  |  |  | M | Liver | Tubular AC |  | >70% |  |  |  |  | 366 | No | Yes |  |
| 6 | M | 57 | P | Sigmoid | Tubular AC | Moderate | >70% | MSI-low | 3 | 11/14 | 1 |  |  | Yes | 60% |
|  |  |  | LR | Sigmoid | Tubular AC | Moderate | >70% |  |  |  |  |  |  | Yes | 80% |
|  |  |  | M | Liver | Tubular AC |  | >70% |  |  |  |  | 0/635 ^(1)^ | No/No | Yes |  |
| 7 | F | 56 | P | Sigmoid | Tubular AC | Moderate | >70% | MSS | 3 | 5/9 | 0 |  |  | No |  |
|  |  |  | M | Liver | Tubular AC |  | >70% |  |  |  |  | 711 | Ap, 6x 5-FU/LV | No |  |
| 8 | M | 73 | P | Coecum | Tubular AC | Moderate | >70% | MSS | 4 | 3/11 | 0 |  |  | No |  |
|  |  |  | M | Mesenterial | Tubular AC |  | >70% |  |  |  |  | 1399 | No | No |  |
| 9 | F | 35 | P | Sigmoid | Tubular AC | Poor | >70% | MSS | 3 | 2/5 | 1 |  |  | No |  |
|  |  |  | M | Ovarian | Tubular AC |  | >70% |  |  |  |  | 48 ^(1)^ | No | No |  |
| 10 | F | 50 | P | Coecum | Tubular AC | Moderate | >70% | MSS | 3 | 0/25 | 1 |  |  | Yes | 50% |
|  |  |  | M | Ovarian | Tubular AC |  | >70% |  |  |  |  | 0 | No | No |  |
| 11 | M | 81 | P | Sigmoid | Tubular AC | Moderate | >70% | failed | 3 | 1/14 | 1 |  |  | No |  |
|  |  |  | M | Liver | Tubular AC |  | >70% |  |  |  |  | 0 | No | No |  |
|  |  |  | M | Stomach | Undifferentiated AC | | >70% |  |  |  |  | 1383 | No | Yes |  |
| 12 | F | 66 | P ^(2)^ | Transversum | Mucinous AC | Moderate | >70% | MSS | 2 | 0/11 | 0 |  |  | No |  |
|  |  |  | M | Liver | Mucinous AC |  | 50% |  |  |  |  | 407 ^(1)^ | No | Yes |  |
|  |  |  | M | Lung | Tubular AC |  | 45% |  |  |  |  | 650 | No | Yes |  |
| 13 | M | 68 | P | Sigmoid | Mucinous AC | Moderate | >70% | MSS | 3 | 1/29 | 0 |  |  | Yes | 60% |
|  |  |  | M | Lung | Mucinous AC |  | 35% |  |  |  |  | 625 | No | No |  |
|  |  |  | M | Thoracic wall | Mucinous AC |  | >70% |  |  |  |  | 1536 | Pal, RTx and 6x xeloda | No |  |
| 14 | F | 66 | P | Coecum | Tubular AC | Moderate | >70% | MSS | 3 | 7/7 | 0 |  |  | Yes | 60% |
|  |  |  | M | Omental | Tubular AC |  | >70% |  |  |  |  | 348 | Ap, 6x 5-FU/LV + Pal, 3x Irinotecan | No |  |
|  |  |  | M | Ovarian | Tubular AC |  | >70% |  |  |  |  | 348 | Ap, 6x 5-FU/LV + Pal, 3x Irinotecan | No |  |
| 15 | F | 77 | P | Sigmoid | Tubular AC | Moderate | >70% | failed | 3 | 0/0 | 0 |  |  | Yes | 65% |
|  |  |  | P | Coecum | Tubular AC | Moderate | >70% | MSS | 3 | 0/14 | 0 |  |  | Yes | 90% |
|  |  |  | M | Liver | Tubular AC |  | >70% |  |  |  |  | 268/432 | No/No | Yes |  |
| 16 | F | 55 | P | Sigmoid | Tubular AC | Moderate | 60% | MSS | 4 | 3/4 | 1 |  |  | Yes | 90% |
|  |  |  | M | Ovarian | Tubular AC |  | >70% |  |  |  |  | 0 | No | Yes |  |
| 18 | M | 76 | P | Rectum | Tubular AC | Moderate | >70% | MSS | 2 | 4/26 | 0 |  |  | Yes | 55% |
|  |  |  | M | Liver | Tubular AC |  | >70% |  |  |  |  | 362 | Ap, 4x Xelox | Yes |  |
| 19 | M | 64 | P | Sigmoid | Tubular AC | Moderate | >70% | MSS | 3 | 9/12 | 0 |  |  | No |  |
|  |  |  | M | Liver | Tubular AC |  | >70% |  |  |  |  | 757 | Ap, 6x 5-FU/LV | Yes |  |
| 20 | M | 72 | LR ^(3)^ | Rectum | Tubular AC | Moderate | >70% | MSS | 4 | n.a. | 1 |  |  | No |  |
|  |  |  | M | Liver | Tubular AC |  | >70% |  |  |  |  | 373 ^(1)^ | Am, 8x Xelox | No |  |
| 21 | M | 70 | P | Rectum | Tubular AC | Moderate | >70% | MSS | 3 | 0/21 | 0 |  |  | Yes | 40% |
|  |  |  | M | Liver | Tubular AC |  | >70% |  |  |  |  | 834 | No | Yes |  |
| 22 | M | 78 | P | Ascendens | Tubular AC | Moderate | >70% | MSS | 4 | 0/8 | 1 |  |  | Yes | 65% |
|  |  |  | M | Liver | Tubular AC |  | >70% |  |  |  |  | 0 | No | No |  |
| 23 | F | 53 | P | Sigmoid | Tubular AC | Moderate | >70% | MSI | 4 | 3/6 | 1 |  |  | No |  |
|  |  |  | M | Ovarian | Tubular AC |  | >70% |  |  |  |  | 0 | No | No |  |
| 24 | M | 65 | P | Transversum | Tubular AC | Poor | >70% | MSS | 4 | 1/1 | 1 |  |  | No |  |
|  |  |  | M | Omental | Tubular AC |  | >70% |  |  |  |  | 0 | No | No |  |
| 25 | M | 62 | P | Coecum | Tubular AC | Moderate | >70% | MSS | 3 | 5/8 | 1 |  |  | No |  |
|  |  |  | M | Omental | Tubular AC |  | 60% |  |  |  |  | 0 | No | No |  |
| 26 | M | 64 | P | Coecum | Tubular AC | Moderate | >70% | MSS | 1 | 8/15 | 1 |  |  | No |  |
|  |  |  | M | Omental | Tubular AC |  | 65% |  |  |  |  | 0 | No | No |  |
| 27 | F | 79 | P | Sigmoid | Tubular AC | Moderate | >70% | MSS | 3 | 14/23 | 1 |  |  | Yes | 50% |
|  |  |  | M | Lymphnode | Tubular AC |  | >70% |  |  |  |  | 0 | No | No |  |
| 28 | M | 74 | P | Coecum | Tubular AC | Moderate | 50% | MSS | 3 | 0/28 | 1 |  |  | Yes | 90% |
|  |  |  | M | Liver | Tubular AC |  | >70% |  |  |  |  | 371 | Apm, Xelox | Yes |  |
| 29 | F | 51 | P | Sigmoid | Tubular AC | Moderate | 55% | MSS | 3 | 0/2 | 1 |  |  | Yes | 80% |
|  |  |  | M | Liver | Tubular AC |  | >70% |  |  |  |  | 526 | No | No |  |
| 30 | F | 39 | P | Rectum | Tubular AC | Moderate | 70% | MSS | 3 | 7/15 | 1 |  |  | Yes | 100% |
|  |  |  | M | Liver | Tubular AC |  | >70% |  |  |  |  | 0 | No | No |  |
| 31 | M | 72 | P | Desecendens | Mucinous AC | Well | 50% | MSS | 3 | 0/2 | 0 |  |  | Yes | 70% |
|  |  |  | M | Liver | Tubular AC |  | >70% |  |  |  |  | 706 | No | No |  |
| 32 | M | 58 | P | Rectum | Tubular AC | Moderate | 70% | MSS | 3 | 4/6 | 0 |  |  | Yes | 80% |
|  |  |  | M | Lung | Tubular AC |  | >70% |  |  |  |  | 3029 | Ap, 5x 5-FU/LV | Yes |  |
| 34 | M | 59 | P | Rectum | Tubular AC | Moderate | >70% | MSS | 3 | 6/8 | 0 |  |  | Yes | 70% |
|  |  |  | M | Liver | Tubular AC |  | >70% |  |  |  |  | 560 | No | Yes |  |
| 35 | F | 53 | P | Ascendens | Tubular AC | Poor | >70% | MSI | 4 | 13/15 | 1 |  |  | No |  |
|  |  |  | M | Lymphnode | Tubular AC |  | >70% |  |  |  |  | 0 | No | No |  |
|  |  |  | M | Lymphnode | Tubular AC |  | >70% |  |  |  |  | 0 | No | No |  |
| 36 | F | 68 | P | Coecum | Tubular AC | Poor | >70% | MSS | 3 | 12/13 | 1 |  |  | No |  |
|  |  |  | M | Lymphnode | Tubular AC |  | >70% |  |  |  |  | 0 | No | No |  |
| 37 | M | 58 | P | Transversum | Mucinous AC | Poor | >70% | MSS | 4 | 6/12 | 1 |  |  | Yes | 85% |
|  |  |  | M | Lymphnode | Signet ring cell carcinoma | | >70% |  |  |  |  | 12 ^(1)^ | No | No |  |
|  |  |  | M | Lymphnode | Signet ring cell carcinoma | | >70% |  |  |  |  | 12 ^(1)^ | No | No |  |
| 38 | F | 63 | P | Sigmoid | Tubular AC | Poor | >70% | MSS | 3 | 4/14/ | 1 |  |  | No |  |
|  |  |  | M | Ovarian | Tubular AC |  | >70% |  |  |  |  | 13 ^(1)^ | No | No |  |
| 39 | F | 84 | P | Sigmoid | Tubular AC | Moderate | >70% | MSS | 3 | 17/19 | 1 |  |  | No |  |
|  |  |  | M | Lymphnode | Tubular AC |  | 65% |  |  |  |  | 0 | No | No |  |
|  |  |  | M | Lymphnode | Tubular AC |  | >70% |  |  |  |  | 0 | No | No |  |
| 40 | M | 40 | P | Descendens | Tubular AC | Moderate | >70% | MSS | 4 | 0/6 | 0 |  |  | No |  |
|  |  |  | M | Liver | Tubular AC |  | >70% |  |  |  |  | 763 | Neo, 7x Folfox | Yes |  |
| *Note: Patient ID 17 and 33 are not included* | | | | |  |  |  |  |  |  |  |  |  |  |  |
| *Age = age at date of diagnosis* | | | | |  |  |  |  |  |  |  |  |  |  |  |
| *Chemotherapy = chemotherapy in between resection of the primary tumor and resection of the metastasis* | | | | | | | | |  |  |  |  |  |  |  |
| *1 Metastasis first* | | | |  |  |  |  |  |  |  |  |  |  |  |  |
| *2 Second primary. T2N0M0 sigmoid 922 days before not frozen* | | | | | | |  |  |  |  |  |  |  |  |  |
| *3 Local recurrence. T3N1M1 sigmoid 426 days before not frozen* | | | | | | |  |  |  |  |  |  |  |  |  |
| *Abbreviations: P, primary colorectal cancer; M, metastasis; LR, local recurrence; AC, Adenocarcinoma; MS status, microsatellite status; MSS, microsatellite stable; MSI, microsatellite instable,, Ap, adjuvant; Am, adjuvant after metastasectomy; Apm, adjuvant after combined resection of primary and metastasis; Neo, neoadjuvant; Pal, palliative* | | | | | | | | | | | | |  |  |  |
|  |  | | | | | | | | | | | |  |  |  |

**Supplementary Table S3. Upregulated tumor specific miRs.** One hundred thirty five miRs with higher expression in tumor tissue compared to normal tissue.

|  | **Geometric mean** | | | | **FDR** | | **Log fold change** | |
| --- | --- | --- | --- | --- | --- | --- | --- | --- |
| **miRNA** | **M** | **MN** | **pCRC** | **PN** | **MN - M** | **PN - pCRC** | **MN - M** | **PN - pCRC** |
| hsa-miR-552-5p | 70,1 | 20,8 | 44,0 | 35,9 | 0,0000 | 0,0000 | -4,25 | -1,28 |
| hsa-miR-552-3p | 146,0 | 38,7 | 71,6 | 48,1 | 0,0000 | 0,0000 | -3,97 | -1,55 |
| hsa-miR-767-5p | 9,3 | 3,4 | 3,6 | 2,8 | 0,0014 | 0,0019 | -3,80 | -2,13 |
| hsa-miR-1246 | 293,0 | 67,9 | 79,0 | 56,7 | 0,0000 | 0,0449 | -3,39 | -0,57 |
| hsa-miR-3937 | 1,7 | 0,9 | 4,0 | 0,2 | 0,0030 | 0,0173 | -3,16 | -2,68 |
| hsa-miR-549a | 8,6 | 2,4 | 4,4 | 0,2 | 0,0000 | 0,0000 | -3,14 | -3,09 |
| hsa-miR-7-5p | 229,0 | 51,9 | 274,0 | 96,5 | 0,0000 | 0,0000 | -3,03 | -1,24 |
| hsa-miR-135b-5p | 247,0 | 46,2 | 191,0 | 13,4 | 0,0000 | 0,0000 | -2,89 | -2,80 |
| hsa-miR-1290 | 8,8 | 3,3 | 2,2 | 1,2 | 0,0001 | 0,0002 | -2,86 | -1,68 |
| hsa-miR-3180-3p | 4,2 | 2,3 | 4,7 | 0,2 | 0,0005 | 0,0001 | -2,83 | -3,13 |
| hsa-miR-183-5p | 3919,0 | 980,0 | 4259,0 | 842,0 | 0,0000 | 0,0000 | -2,66 | -1,42 |
| hsa-miR-4652-5p | 1,4 | 0,4 | 1,2 | 0,2 | 0,0000 | 0,0022 | -2,62 | -1,46 |
| hsa-miR-7641 | 19,8 | 7,0 | 4,5 | 1,7 | 0,0000 | 0,0001 | -2,57 | -1,68 |
| hsa-miR-135b-3p | 17,7 | 5,2 | 16,2 | 0,5 | 0,0000 | 0,0000 | -2,54 | -3,55 |
| hsa-chr11_27716-3p | 1,2 | 0,7 | 1,0 | 0,3 | 0,0000 | 0,0004 | -2,52 | -1,40 |
| hsa-miR-4713-5p | 1,8 | 0,6 | 1,3 | 0,5 | 0,0000 | 0,0021 | -2,51 | -1,07 |
| hsa-miR-3180-5p | 1,6 | 0,8 | 1,4 | 0,2 | 0,0054 | 0,0064 | -2,50 | -2,03 |
| hsa-miR-183-3p | 20,7 | 8,0 | 20,5 | 5,8 | 0,0000 | 0,0000 | -2,39 | -1,45 |
| hsa-miR-4664-3p | 2,0 | 1,1 | 2,5 | 0,4 | 0,0000 | 0,0005 | -2,37 | -1,75 |
| hsa-miR-182-5p | 36199,0 | 9599,0 | 29399,0 | 9189,0 | 0,0000 | 0,0000 | -2,29 | -1,28 |
| hsa-miR-592 | 111,0 | 94,8 | 135,0 | 67,8 | 0,0000 | 0,0000 | -2,27 | -1,59 |
| hsa-miR-6087 | 6,4 | 1,9 | 2,3 | 1,1 | 0,0001 | 0,0017 | -2,24 | -1,20 |
| hsa-miR-1269a | 18,9 | 9,6 | 6,0 | 1,9 | 0,0010 | 0,0004 | -2,20 | -2,19 |
| hsa-miR-96-5p | 155,0 | 46,8 | 217,0 | 44,1 | 0,0000 | 0,0000 | -2,17 | -1,30 |
| hsa-miR-509-3p | 21,1 | 6,0 | 5,6 | 1,6 | 0,0010 | 0,0000 | -2,16 | -1,36 |
| hsa-miR-767-3p | 0,8 | 0,2 | 0,5 | 0,0 | 0,0120 | 0,0266 | -2,16 | -1,38 |
| hsa-miR-7974 | 29,7 | 14,6 | 33,2 | 4,9 | 0,0000 | 0,0000 | -2,15 | -2,27 |
| hsa-miR-508-3p | 6,9 | 2,3 | 1,3 | 0,4 | 0,0042 | 0,0000 | -1,97 | -1,86 |
| hsa-miR-224-5p | 745,0 | 396,0 | 647,0 | 191,0 | 0,0000 | 0,0000 | -1,90 | -1,74 |
| hsa-miR-3651 | 8,1 | 4,8 | 3,9 | 2,6 | 0,0000 | 0,0036 | -1,88 | -1,12 |
| hsa-chr8_21912-3p | 199,0 | 137,0 | 175,0 | 92,1 | 0,0000 | 0,0124 | -1,87 | -0,91 |
| hsa-chr8_22338-3p | 0,7 | 0,5 | 1,2 | 0,2 | 0,0043 | 0,0014 | -1,85 | -1,30 |
| hsa-miR-301b | 205,0 | 80,6 | 186,0 | 82,6 | 0,0000 | 0,0000 | -1,83 | -1,09 |
| hsa-chr17_38208-5p | 1,0 | 0,5 | 1,1 | 0,1 | 0,0098 | 0,0000 | -1,81 | -2,09 |
| hsa-chr9_22918-5p | 0,8 | 0,3 | 0,6 | 0,1 | 0,0047 | 0,0109 | -1,74 | -1,11 |
| hsa-miR-4697-3p | 3,1 | 1,1 | 3,0 | 1,0 | 0,0076 | 0,0274 | -1,74 | -0,67 |
| hsa-miR-1910-5p | 1,3 | 0,5 | 1,2 | 0,3 | 0,0061 | 0,0098 | -1,73 | -1,24 |
| hsa-chr16_35996-5p | 0,3 | 0,1 | 0,4 | 0,2 | 0,0053 | 0,0405 | -1,72 | -0,82 |
| hsa-miR-584-5p | 288,0 | 126,0 | 179,0 | 52,0 | 0,0000 | 0,0000 | -1,71 | -1,68 |
| hsa-miR-935 | 4,7 | 2,3 | 2,8 | 0,8 | 0,0006 | 0,0029 | -1,65 | -1,60 |
| hsa-chr7_19202-5p | 7,3 | 4,6 | 9,9 | 1,7 | 0,0000 | 0,0000 | -1,65 | -1,96 |
| hsa-chr8_22327-5p | 0,3 | 0,3 | 0,3 | 0,1 | 0,0157 | 0,0453 | -1,61 | -0,70 |
| hsa-miR-4449 | 9,0 | 4,8 | 6,7 | 3,6 | 0,0027 | 0,0023 | -1,61 | -0,98 |
| hsa-miR-4661-5p | 7,2 | 4,1 | 4,1 | 2,4 | 0,0000 | 0,0007 | -1,60 | -0,95 |
| hsa-miR-1247-5p | 237,0 | 165,0 | 404,0 | 98,8 | 0,0000 | 0,0000 | -1,60 | -1,42 |
| hsa-miR-10a-3p | 240,0 | 134,0 | 275,0 | 192,0 | 0,0000 | 0,0137 | -1,58 | -0,38 |
| hsa-miR-6516-5p | 4,1 | 2,2 | 3,1 | 2,2 | 0,0001 | 0,0031 | -1,58 | -0,67 |
| hsa-chr1_2265-3p | 2,5 | 1,6 | 2,7 | 1,1 | 0,0001 | 0,0011 | -1,56 | -1,01 |
| hsa-miR-1276 | 3,5 | 1,5 | 3,6 | 1,5 | 0,0001 | 0,0078 | -1,56 | -0,81 |
| hsa-miR-1226-5p | 0,6 | 0,5 | 0,4 | 0,2 | 0,0058 | 0,0104 | -1,56 | -1,18 |
| hsa-miR-3200-3p | 17,1 | 10,0 | 16,7 | 6,8 | 0,0000 | 0,0000 | -1,55 | -1,22 |
| hsa-miR-4745-5p | 0,6 | 0,3 | 0,6 | 0,2 | 0,0107 | 0,0337 | -1,54 | -0,96 |
| hsa-chr5_13669-3p | 0,8 | 0,7 | 1,2 | 0,7 | 0,0078 | 0,0272 | -1,53 | -0,76 |
| hsa-miR-18a-5p | 337,0 | 184,0 | 266,0 | 150,0 | 0,0000 | 0,0001 | -1,53 | -0,84 |
| hsa-miR-937-3p | 6,3 | 2,9 | 5,8 | 1,2 | 0,0003 | 0,0000 | -1,53 | -1,51 |
| hsa-chr17_38309-3p | 2,4 | 1,8 | 2,2 | 1,0 | 0,0029 | 0,0007 | -1,52 | -1,07 |
| hsa-miR-18a-3p | 37,1 | 17,9 | 37,2 | 15,0 | 0,0000 | 0,0000 | -1,52 | -0,98 |
| hsa-miR-4326 | 13,0 | 7,0 | 17,2 | 11,1 | 0,0026 | 0,0000 | -1,47 | -0,75 |
| hsa-miR-3177-3p | 2,4 | 1,3 | 2,2 | 0,8 | 0,0010 | 0,0008 | -1,44 | -0,85 |
| hsa-miR-320d | 41,0 | 20,9 | 27,0 | 20,7 | 0,0000 | 0,0482 | -1,41 | -0,31 |
| hsa-miR-466 | 0,2 | 0,2 | 0,0 | 0,0 | 0,0304 | 0,0215 | -1,39 | -1,22 |
| hsa-miR-95-5p | 12,0 | 7,3 | 8,1 | 5,0 | 0,0000 | 0,0023 | -1,37 | -0,96 |
| hsa-miR-5094 | 0,9 | 0,5 | 0,9 | 0,2 | 0,0134 | 0,0044 | -1,37 | -1,41 |
| hsa-chr13_31956-3p | 0,2 | 0,2 | 0,3 | 0,0 | 0,0263 | 0,0235 | -1,36 | -1,07 |
| hsa-miR-3189-3p | 0,4 | 0,1 | 0,4 | 0,0 | 0,0290 | 0,0060 | -1,31 | -1,46 |
| hsa-chr13_31997-5p | 0,3 | 0,1 | 0,4 | 0,0 | 0,0272 | 0,0218 | -1,28 | -1,18 |
| hsa-miR-940 | 14,0 | 8,8 | 13,6 | 9,2 | 0,0000 | 0,0006 | -1,28 | -0,62 |
| hsa-miR-3679-5p | 4,1 | 2,6 | 3,6 | 1,1 | 0,0004 | 0,0000 | -1,26 | -1,50 |
| hsa-miR-550a-5p | 22,3 | 13,1 | 26,5 | 13,5 | 0,0000 | 0,0005 | -1,23 | -0,60 |
| hsa-miR-181d-5p | 528,0 | 304,0 | 473,0 | 230,0 | 0,0000 | 0,0000 | -1,22 | -0,97 |
| hsa-miR-95-3p | 129,0 | 93,0 | 116,0 | 78,3 | 0,0000 | 0,0001 | -1,21 | -0,72 |
| hsa-miR-21-5p | 256999,0 | 156999,0 | 232999,0 | 128999,0 | 0,0000 | 0,0000 | -1,18 | -0,79 |
| hsa-miR-208b-3p | 0,7 | 0,2 | 0,4 | 0,1 | 0,0211 | 0,0398 | -1,17 | -0,87 |
| hsa-miR-320b | 330,0 | 202,0 | 220,0 | 214,0 | 0,0000 | 0,0369 | -1,17 | -0,15 |
| hsa-miR-182-3p | 1,6 | 0,8 | 1,3 | 0,7 | 0,0044 | 0,0248 | -1,16 | -0,77 |
| hsa-miR-335-3p | 1679,0 | 1079,0 | 1809,0 | 695,0 | 0,0000 | 0,0000 | -1,15 | -1,04 |
| hsa-miR-3176 | 23,9 | 13,0 | 24,7 | 9,0 | 0,0000 | 0,0000 | -1,15 | -1,07 |
| hsa-miR-877-5p | 42,6 | 25,1 | 42,5 | 21,3 | 0,0002 | 0,0000 | -1,15 | -0,96 |
| hsa-miR-6886-5p | 0,3 | 0,3 | 0,6 | 0,1 | 0,0347 | 0,0100 | -1,14 | -1,30 |
| hsa-miR-17-5p | 2549,0 | 1599,0 | 2139,0 | 1139,0 | 0,0000 | 0,0000 | -1,12 | -0,92 |
| hsa-miR-3144-3p | 1,0 | 0,8 | 0,3 | 0,2 | 0,0482 | 0,0009 | -1,10 | -1,82 |
| hsa-miR-421 | 625,0 | 350,0 | 490,0 | 305,0 | 0,0000 | 0,0000 | -1,10 | -0,59 |
| hsa-miR-21-3p | 14899,0 | 8409,0 | 13099,0 | 5049,0 | 0,0000 | 0,0000 | -1,10 | -1,11 |
| hsa-miR-1254 | 6,6 | 2,9 | 8,3 | 2,6 | 0,0006 | 0,0003 | -1,10 | -1,19 |
| hsa-miR-4435 | 1,5 | 1,0 | 1,9 | 0,3 | 0,0192 | 0,0001 | -1,09 | -1,69 |
| hsa-miR-452-5p | 220,0 | 150,0 | 195,0 | 130,0 | 0,0000 | 0,0000 | -1,09 | -0,76 |
| hsa-miR-1229-3p | 1,6 | 0,9 | 1,6 | 0,8 | 0,0138 | 0,0175 | -1,08 | -0,84 |
| hsa-miR-7705 | 41,4 | 24,0 | 43,8 | 29,2 | 0,0005 | 0,0088 | -1,08 | -0,43 |
| hsa-miR-1268a | 14,4 | 11,9 | 20,9 | 13,1 | 0,0024 | 0,0003 | -1,08 | -0,79 |
| hsa-miR-19a-5p | 9,1 | 5,9 | 9,9 | 4,2 | 0,0003 | 0,0003 | -1,07 | -0,96 |
| hsa-miR-6753-3p | 0,6 | 0,2 | 0,7 | 0,1 | 0,0329 | 0,0256 | -1,06 | -1,13 |
| hsa-miR-4517 | 2,8 | 1,6 | 2,4 | 0,8 | 0,0125 | 0,0001 | -1,04 | -1,24 |
| hsa-miR-4488 | 13,6 | 9,4 | 8,4 | 6,3 | 0,0201 | 0,0103 | -1,04 | -0,97 |
| hsa-miR-501-5p | 17,2 | 12,2 | 15,6 | 13,3 | 0,0001 | 0,0493 | -1,04 | -0,30 |
| hsa-miR-4485 | 33,1 | 26,6 | 27,7 | 10,7 | 0,0038 | 0,0001 | -1,04 | -1,33 |
| hsa-miR-2467-5p | 25,3 | 12,4 | 28,3 | 12,4 | 0,0000 | 0,0000 | -1,03 | -0,66 |
| hsa-miR-1268b | 16,3 | 13,3 | 22,0 | 14,0 | 0,0025 | 0,0003 | -1,03 | -0,78 |
| hsa-miR-20a-5p | 3629,0 | 2439,0 | 3069,0 | 1829,0 | 0,0000 | 0,0000 | -1,02 | -0,88 |
| hsa-miR-222-3p | 3419,0 | 1809,0 | 3609,0 | 2429,0 | 0,0011 | 0,0012 | -0,99 | -0,44 |
| hsa-miR-93-5p | 6199,0 | 4159,0 | 5359,0 | 3809,0 | 0,0000 | 0,0000 | -0,96 | -0,53 |
| hsa-miR-4454 | 23,8 | 20,5 | 32,6 | 15,1 | 0,0149 | 0,0000 | -0,94 | -1,09 |
| hsa-miR-2276-3p | 1,4 | 0,7 | 1,4 | 0,7 | 0,0102 | 0,0246 | -0,94 | -0,73 |
| hsa-miR-339-5p | 211,0 | 143,0 | 199,0 | 157,0 | 0,0000 | 0,0042 | -0,93 | -0,34 |
| hsa-miR-941 | 1539,0 | 1029,0 | 1749,0 | 923,0 | 0,0000 | 0,0189 | -0,92 | -0,45 |
| hsa-miR-19a-3p | 951,0 | 576,0 | 734,0 | 436,0 | 0,0001 | 0,0002 | -0,91 | -0,69 |
| hsa-miR-708-5p | 55,5 | 37,6 | 120,0 | 30,7 | 0,0045 | 0,0000 | -0,91 | -1,36 |
| hsa-miR-181c-5p | 3839,0 | 2649,0 | 3109,0 | 2519,0 | 0,0007 | 0,0004 | -0,91 | -0,54 |
| hsa-miR-93-3p | 36,6 | 25,0 | 45,9 | 32,1 | 0,0001 | 0,0442 | -0,90 | -0,23 |
| hsa-chr10_24674-5p | 16,1 | 14,0 | 24,9 | 11,3 | 0,0016 | 0,0001 | -0,89 | -0,85 |
| hsa-miR-7706 | 125,0 | 81,2 | 133,0 | 79,8 | 0,0039 | 0,0201 | -0,88 | -0,40 |
| hsa-miR-181c-3p | 317,0 | 205,0 | 256,0 | 168,0 | 0,0003 | 0,0000 | -0,87 | -0,68 |
| hsa-miR-532-5p | 2819,0 | 1769,0 | 2389,0 | 1719,0 | 0,0000 | 0,0119 | -0,83 | -0,36 |
| hsa-miR-671-5p | 27,0 | 18,9 | 34,4 | 22,5 | 0,0000 | 0,0006 | -0,81 | -0,43 |
| hsa-miR-188-5p | 37,3 | 27,1 | 37,7 | 33,4 | 0,0002 | 0,0186 | -0,80 | -0,43 |
| hsa-miR-25-5p | 25,8 | 20,4 | 34,3 | 16,2 | 0,0032 | 0,0000 | -0,79 | -0,73 |
| hsa-miR-92a-1-5p | 34,0 | 32,3 | 37,3 | 15,2 | 0,0028 | 0,0000 | -0,78 | -1,25 |
| hsa-miR-20a-3p | 17,9 | 11,4 | 12,3 | 8,4 | 0,0008 | 0,0011 | -0,78 | -0,80 |
| hsa-miR-454-5p | 20,6 | 11,3 | 20,7 | 13,8 | 0,0012 | 0,0027 | -0,77 | -0,41 |
| hsa-miR-301a-3p | 973,0 | 600,0 | 638,0 | 577,0 | 0,0053 | 0,0309 | -0,77 | -0,41 |
| hsa-miR-1292-5p | 1,6 | 0,9 | 1,8 | 0,6 | 0,0161 | 0,0046 | -0,77 | -1,04 |
| hsa-miR-503-5p | 8,9 | 6,3 | 7,7 | 1,3 | 0,0200 | 0,0000 | -0,75 | -1,90 |
| hsa-miR-3609 | 12,5 | 9,0 | 11,8 | 5,1 | 0,0017 | 0,0038 | -0,72 | -0,91 |
| hsa-miR-19b-3p | 3819,0 | 2739,0 | 2679,0 | 1859,0 | 0,0021 | 0,0002 | -0,69 | -0,65 |
| hsa-miR-92b-3p | 3529,0 | 2329,0 | 5749,0 | 3049,0 | 0,0156 | 0,0166 | -0,65 | -0,39 |
| hsa-miR-106b-3p | 831,0 | 571,0 | 720,0 | 491,0 | 0,0000 | 0,0000 | -0,65 | -0,40 |
| hsa-miR-708-3p | 38,9 | 28,4 | 68,8 | 18,1 | 0,0034 | 0,0000 | -0,65 | -1,43 |
| hsa-miR-1285-5p | 3,4 | 2,6 | 2,8 | 1,7 | 0,0381 | 0,0280 | -0,63 | -0,51 |
| hsa-miR-98-5p | 5709,0 | 4489,0 | 6519,0 | 4219,0 | 0,0013 | 0,0408 | -0,63 | -0,26 |
| hsa-miR-6516-3p | 2,9 | 2,3 | 2,5 | 1,8 | 0,0344 | 0,0385 | -0,62 | -0,35 |
| hsa-miR-130b-3p | 630,0 | 449,0 | 701,0 | 423,0 | 0,0004 | 0,0002 | -0,62 | -0,45 |
| hsa-miR-92a-3p | 99499,0 | 73699,0 | 80099,0 | 40799,0 | 0,0006 | 0,0000 | -0,60 | -0,90 |
| hsa-miR-23a-3p | 3519,0 | 2829,0 | 3419,0 | 2859,0 | 0,0036 | 0,0191 | -0,58 | -0,24 |
| hsa-miR-98-3p | 50,1 | 35,3 | 52,1 | 39,6 | 0,0068 | 0,0225 | -0,56 | -0,32 |
| hsa-miR-431-5p | 25,4 | 20,2 | 30,9 | 10,5 | 0,0213 | 0,0000 | -0,53 | -1,12 |
| hsa-miR-25-3p | 10099,0 | 8349,0 | 9449,0 | 6099,0 | 0,0148 | 0,0000 | -0,37 | -0,46 |
|  |  |  |  |  |  |  |  |  |
| Expression level is noted as mean geometic value. | | |  |  |  |  |  |  |
| Fold change is noted as natural logarithm | | |  |  |  |  |  |  |
| False discovery rate (FDR) was estimated using the Bayesian FDR estimate | | | |  |  |  |  |  |
| Abbreviations: M = metastases, pCRC = primary colorectal cancer, MN = normal extracolonic tissue, PN = normal colorectal mucosa | | | | | | | |  |

**Supplementary Table S4. Down regulated tumor specific miRs. Eighty-seven miRs with lower expression in tumor tissue compared to normal tissue.**

|  | **Geometric mean** | | | | **FDR** | | **Log fold change** | |
| --- | --- | --- | --- | --- | --- | --- | --- | --- |
| **miRNA** | **M** | **MN** | **pCRC** | **PN** | **MN - M** | **PN - pCRC** | **MN - M** | **PN - pCRC** |
| hsa-miR-490-3p | 0,9 | 9,0 | 4,0 | 15,6 | 0,0000 | 0,0024 | 2,61 | 1,42 |
| hsa-miR-139-5p | 26,0 | 98,1 | 27,6 | 124,0 | 0,0000 | 0,0000 | 2,61 | 1,48 |
| hsa-miR-4524a-3p | 0,3 | 1,7 | 0,3 | 1,0 | 0,0000 | 0,0041 | 2,54 | 0,91 |
| hsa-miR-490-5p | 0,2 | 0,7 | 1,1 | 2,4 | 0,0001 | 0,0114 | 2,20 | 1,31 |
| hsa-miR-488-3p | 0,5 | 1,3 | 0,0 | 2,7 | 0,0000 | 0,0000 | 2,13 | 2,98 |
| hsa-miR-451a | 1589,0 | 4279,0 | 1219,0 | 3529,0 | 0,0000 | 0,0000 | 2,11 | 0,95 |
| hsa-miR-139-3p | 1,0 | 3,5 | 1,0 | 4,9 | 0,0000 | 0,0000 | 2,02 | 1,42 |
| hsa-miR-551b-3p | 1,1 | 2,7 | 0,5 | 6,3 | 0,0000 | 0,0000 | 2,02 | 1,85 |
| hsa-miR-3622a-5p | 0,4 | 0,8 | 0,7 | 2,7 | 0,0000 | 0,0000 | 1,98 | 1,90 |
| hsa-miR-30c-2-3p | 16,8 | 45,6 | 12,4 | 33,0 | 0,0000 | 0,0000 | 1,90 | 0,66 |
| hsa-miR-4662a-5p | 10,7 | 19,7 | 12,0 | 27,4 | 0,0000 | 0,0053 | 1,89 | 0,70 |
| hsa-miR-378e | 2,1 | 4,1 | 2,7 | 4,7 | 0,0000 | 0,0012 | 1,89 | 0,82 |
| hsa-miR-216b-5p | 0,3 | 1,1 | 0,1 | 0,9 | 0,0001 | 0,0010 | 1,84 | 1,61 |
| hsa-miR-30a-3p | 232,0 | 510,0 | 164,0 | 472,0 | 0,0000 | 0,0000 | 1,78 | 0,80 |
| hsa-miR-30a-5p | 9449,0 | 22399,0 | 8549,0 | 22099,0 | 0,0000 | 0,0000 | 1,73 | 0,82 |
| hsa-miR-144-3p | 160,0 | 408,0 | 149,0 | 368,0 | 0,0000 | 0,0001 | 1,72 | 0,88 |
| hsa-miR-144-5p | 103,0 | 256,0 | 69,7 | 216,0 | 0,0000 | 0,0001 | 1,66 | 0,88 |
| hsa-miR-497-3p | 2,4 | 4,3 | 2,5 | 10,8 | 0,0000 | 0,0000 | 1,50 | 1,60 |
| hsa-miR-363-3p | 77,5 | 140,0 | 92,4 | 229,0 | 0,0000 | 0,0000 | 1,45 | 1,21 |
| hsa-miR-195-5p | 612,0 | 1359,0 | 814,0 | 2549,0 | 0,0000 | 0,0000 | 1,43 | 1,03 |
| hsa-miR-195-3p | 25,3 | 58,0 | 31,9 | 93,9 | 0,0000 | 0,0000 | 1,34 | 0,97 |
| hsa-miR-1468-5p | 45,9 | 80,2 | 37,0 | 71,4 | 0,0000 | 0,0030 | 1,32 | 0,52 |
| hsa-miR-3614-3p | 0,5 | 1,1 | 0,6 | 1,5 | 0,0007 | 0,0040 | 1,30 | 0,77 |
| hsa-miR-29c-3p | 662,0 | 1259,0 | 648,0 | 2309,0 | 0,0000 | 0,0000 | 1,28 | 1,10 |
| hsa-miR-497-5p | 346,0 | 761,0 | 425,0 | 1439,0 | 0,0000 | 0,0000 | 1,27 | 1,08 |
| hsa-miR-4424 | 0,5 | 1,2 | 0,8 | 2,3 | 0,0015 | 0,0000 | 1,26 | 1,10 |
| hsa-miR-5695 | 0,3 | 0,5 | 0,6 | 1,0 | 0,0089 | 0,0035 | 1,26 | 0,97 |
| hsa-miR-30c-1-3p | 27,0 | 45,7 | 29,8 | 60,3 | 0,0000 | 0,0000 | 1,26 | 0,77 |
| hsa-miR-29c-5p | 30,7 | 57,5 | 27,3 | 83,1 | 0,0000 | 0,0000 | 1,25 | 0,88 |
| hsa-miR-378h | 0,9 | 1,6 | 1,1 | 4,0 | 0,0008 | 0,0000 | 1,24 | 1,36 |
| hsa-miR-548ba | 1,4 | 1,7 | 2,3 | 6,3 | 0,0229 | 0,0017 | 1,20 | 1,21 |
| hsa-miR-378i | 112,0 | 192,0 | 123,0 | 525,0 | 0,0000 | 0,0000 | 1,18 | 1,49 |
| hsa-miR-6507-5p | 0,1 | 0,5 | 0,4 | 2,0 | 0,0319 | 0,0008 | 1,18 | 0,90 |
| hsa-miR-30e-3p | 831,0 | 1339,0 | 653,0 | 1629,0 | 0,0000 | 0,0000 | 1,15 | 0,75 |
| hsa-miR-30c-5p | 2429,0 | 3919,0 | 2579,0 | 5779,0 | 0,0000 | 0,0000 | 1,14 | 0,79 |
| hsa-miR-22-3p | 64599,0 | 111999,0 | 68999,0 | 106999,0 | 0,0000 | 0,0015 | 1,11 | 0,40 |
| hsa-miR-101-5p | 32,3 | 52,5 | 35,5 | 56,5 | 0,0000 | 0,0094 | 1,05 | 0,41 |
| hsa-miR-1265 | 0,0 | 0,1 | 0,0 | 2,2 | 0,0454 | 0,0000 | 1,04 | 2,95 |
| hsa-miR-3611 | 1,2 | 1,8 | 1,5 | 2,5 | 0,0060 | 0,0116 | 1,01 | 0,51 |
| hsa-miR-26b-5p | 11999,0 | 17599,0 | 9829,0 | 20999,0 | 0,0000 | 0,0000 | 0,98 | 0,62 |
| hsa-miR-4999-5p | 1,5 | 2,1 | 1,5 | 3,1 | 0,0065 | 0,0026 | 0,97 | 0,69 |
| hsa-miR-30e-5p | 12199,0 | 18499,0 | 11199,0 | 26699,0 | 0,0000 | 0,0000 | 0,95 | 0,77 |
| hsa-miR-628-5p | 20,8 | 27,6 | 19,3 | 32,3 | 0,0000 | 0,0001 | 0,95 | 0,56 |
| hsa-miR-378d | 186,0 | 311,0 | 177,0 | 760,0 | 0,0000 | 0,0000 | 0,94 | 1,33 |
| hsa-miR-24-1-5p | 9,4 | 16,4 | 10,9 | 17,7 | 0,0000 | 0,0261 | 0,93 | 0,32 |
| hsa-miR-511-3p | 2,6 | 5,0 | 2,1 | 6,5 | 0,0065 | 0,0000 | 0,92 | 0,91 |
| hsa-miR-570-5p | 0,6 | 1,0 | 0,3 | 1,3 | 0,0268 | 0,0049 | 0,91 | 0,79 |
| hsa-miR-548ai | 0,6 | 1,0 | 0,3 | 1,3 | 0,0270 | 0,0050 | 0,91 | 0,79 |
| hsa-miR-340-3p | 24,6 | 29,8 | 20,2 | 33,1 | 0,0000 | 0,0028 | 0,90 | 0,44 |
| hsa-miR-574-3p | 522,0 | 844,0 | 580,0 | 914,0 | 0,0000 | 0,0033 | 0,87 | 0,39 |
| hsa-miR-3912-3p | 12,7 | 16,0 | 10,4 | 18,3 | 0,0001 | 0,0016 | 0,86 | 0,48 |
| hsa-miR-548h-5p | 5,7 | 7,6 | 4,7 | 8,7 | 0,0071 | 0,0014 | 0,86 | 0,55 |
| hsa-miR-511-5p | 7,5 | 14,7 | 5,5 | 19,4 | 0,0090 | 0,0000 | 0,86 | 0,87 |
| hsa-miR-133a-3p | 61,5 | 159,0 | 154,0 | 1029,0 | 0,0140 | 0,0000 | 0,82 | 1,88 |
| hsa-miR-378a-5p | 68,4 | 110,0 | 72,5 | 363,0 | 0,0003 | 0,0000 | 0,82 | 1,42 |
| hsa-chr3_8875-3p | 1,7 | 2,4 | 1,9 | 5,7 | 0,0209 | 0,0000 | 0,80 | 1,07 |
| hsa-miR-378f | 32,9 | 58,3 | 24,0 | 146,0 | 0,0010 | 0,0000 | 0,79 | 1,37 |
| hsa-miR-548h-3p | 6,3 | 8,0 | 5,2 | 8,8 | 0,0005 | 0,0312 | 0,78 | 0,35 |
| hsa-miR-548z | 6,4 | 7,9 | 5,3 | 9,2 | 0,0007 | 0,0213 | 0,77 | 0,39 |
| hsa-miR-887-5p | 2,6 | 3,3 | 3,6 | 5,8 | 0,0143 | 0,0013 | 0,74 | 0,61 |
| hsa-miR-548ar-5p | 3,8 | 4,6 | 4,0 | 6,6 | 0,0119 | 0,0009 | 0,74 | 0,65 |
| hsa-miR-6503-5p | 1,1 | 1,4 | 0,8 | 2,4 | 0,0072 | 0,0004 | 0,73 | 1,13 |
| hsa-miR-145-3p | 293,0 | 512,0 | 674,0 | 1049,0 | 0,0020 | 0,0079 | 0,72 | 0,62 |
| hsa-miR-342-5p | 15,1 | 19,6 | 12,0 | 28,5 | 0,0050 | 0,0000 | 0,71 | 0,66 |
| hsa-miR-616-5p | 6,0 | 7,4 | 5,2 | 9,3 | 0,0018 | 0,0020 | 0,71 | 0,45 |
| hsa-miR-328-3p | 51,7 | 62,3 | 50,5 | 73,2 | 0,0000 | 0,0002 | 0,69 | 0,45 |
| hsa-miR-378a-3p | 13399,0 | 19899,0 | 13499,0 | 59299,0 | 0,0002 | 0,0000 | 0,67 | 1,42 |
| hsa-miR-5690 | 3,0 | 4,0 | 2,8 | 6,6 | 0,0132 | 0,0000 | 0,67 | 0,82 |
| hsa-miR-30b-5p | 3859,0 | 4839,0 | 2949,0 | 5789,0 | 0,0011 | 0,0126 | 0,66 | 0,41 |
| hsa-miR-136-5p | 70,3 | 134,0 | 93,8 | 195,0 | 0,0063 | 0,0131 | 0,66 | 0,45 |
| hsa-miR-26b-3p | 52,0 | 71,5 | 47,2 | 88,8 | 0,0004 | 0,0006 | 0,64 | 0,50 |
| hsa-miR-548g-5p | 4,7 | 5,8 | 4,5 | 8,3 | 0,0375 | 0,0003 | 0,64 | 0,68 |
| hsa-miR-29b-2-5p | 5,5 | 7,6 | 4,3 | 10,5 | 0,0286 | 0,0000 | 0,62 | 0,65 |
| hsa-miR-548c-3p | 2,0 | 2,7 | 1,4 | 4,1 | 0,0194 | 0,0001 | 0,62 | 0,78 |
| hsa-miR-628-3p | 8,7 | 12,4 | 14,4 | 17,3 | 0,0016 | 0,0379 | 0,62 | 0,30 |
| hsa-miR-548aj-5p | 4,8 | 5,7 | 4,5 | 8,3 | 0,0416 | 0,0002 | 0,61 | 0,68 |
| hsa-miR-140-5p | 70,2 | 101,0 | 72,3 | 127,0 | 0,0042 | 0,0182 | 0,60 | 0,37 |
| hsa-miR-378c | 729,0 | 1049,0 | 665,0 | 2889,0 | 0,0033 | 0,0000 | 0,60 | 1,33 |
| hsa-miR-6503-3p | 5,1 | 8,3 | 4,8 | 11,2 | 0,0041 | 0,0052 | 0,60 | 0,54 |
| hsa-miR-33b-5p | 87,5 | 120,0 | 97,6 | 141,0 | 0,0088 | 0,0429 | 0,60 | 0,30 |
| hsa-miR-28-5p | 1339,0 | 1929,0 | 1679,0 | 3079,0 | 0,0000 | 0,0000 | 0,59 | 0,67 |
| hsa-miR-548x-5p | 4,6 | 5,6 | 4,4 | 8,2 | 0,0460 | 0,0002 | 0,59 | 0,67 |
| hsa-miR-422a | 2,7 | 4,7 | 3,4 | 15,5 | 0,0246 | 0,0000 | 0,57 | 1,44 |
| hsa-miR-381-3p | 538,0 | 864,0 | 547,0 | 1129,0 | 0,0126 | 0,0054 | 0,56 | 0,45 |
| hsa-miR-326 | 39,4 | 45,1 | 34,2 | 57,1 | 0,0027 | 0,0000 | 0,56 | 0,59 |
| hsa-miR-140-3p | 1999,0 | 2649,0 | 1709,0 | 2829,0 | 0,0028 | 0,0259 | 0,56 | 0,27 |
| hsa-miR-15a-5p | 1339,0 | 1609,0 | 1209,0 | 1959,0 | 0,0428 | 0,0047 | 0,34 | 0,38 |
|  |  |  |  |  |  |  |  |  |
| Expression level is noted as mean geometic value. | | |  |  |  |  |  |  |
| Fold change is noted as natural logarithm | | |  |  |  |  |  |  |
| False discovery rate (FDR) was estimated using the Bayesian FDR estimate | | | | |  |  |  |  |
| Abbreviations: M = metastases, pCRC = primary colorectal cancer, MN = normal extracolonic tissue, PN = normal colorectal mucosa | | | | | | | |  |
